# Programming Thermochromic Liquid Crystal Hetero-Oligomers for Near-Infrared Reflectors: Unequal Incorporation of Similar Reactive Mesogens in Thiol-ene Oligomers

**DOI:** 10.1021/acs.macromol.2c02041

**Published:** 2022-12-27

**Authors:** Henk Sentjens, Augustinus J.J. Kragt, Johan Lub, Mart D.T. Claessen, Vera E. Buurman, Joris Schreppers, Henk A. Gongriep, Albert P.H.J. Schenning

**Affiliations:** † Laboratory of Stimuli-Responsive Functional Materials and Devices (SFD), Department of Chemical Engineering and Chemistry, Eindhoven University of Technology (TU/e), P.O. Box 513, 5600 MBEindhoven, The Netherlands; ‡ Institute for Complex Molecular Systems, Eindhoven University of Technology (TU/e), P.O. Box 513, 5600 MBEindhoven, The Netherlands; § Faculty of Architecture, Delft University of Technology, Julianalaan 134, 2628 BLDelft, The Netherlands; ∥ ClimAd Technology, Valkenaerhof 68, 6538 TENijmegen, The Netherlands

## Abstract

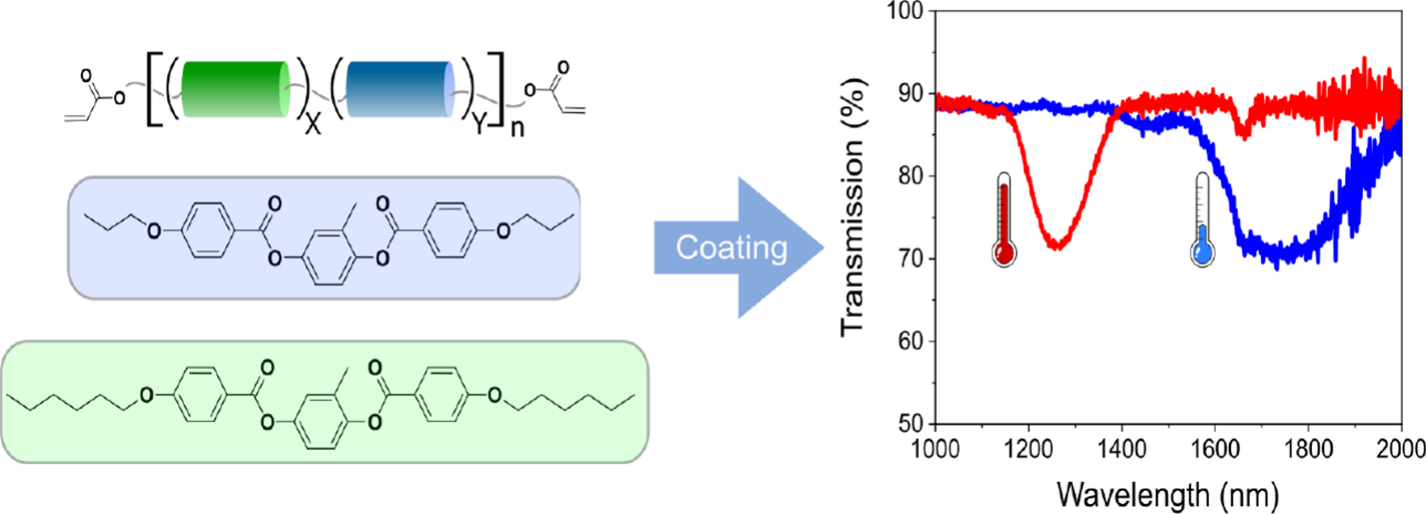

Cholesteric liquid crystal oligomers are widely researched for their interesting thermochromic properties. However, structure–property relationships to program the thermochromic properties of these oligomers have been rarely reported. In this work, we use the versatile thiol-ene click reaction to synthesize a series of hetero-oligomers and study the impact of different compositions on the thermochromic behavior of the resulting material. Characterization of the oligomers shows significantly different rates of reaction for the monomers despite their very similar structures, which leads to oligomer compositions that do not match the original reaction feed. The oligomers are then used to produce thin near-infrared reflecting coatings. The best-performing thermochromic reflector has a room-temperature reflection band that shifts a total of 510 nanometers upon heating to 120 °C. The shift is repeatable for up to 10 times with no appreciable degradation. The room temperature reflection of the coatings is shown to be tunable not only by adjusting the chiral dopant concentration but also by the ratio of the monomers. Finally, we show that the oligomers can be chemically modified by making their reactive end groups undergo a reaction with monothiol compounds. These modifications allow for further fine-tuning of liquid crystal oligomers for heat-regulating window films, for example.

## Introduction

Photonic crystals are nanomaterials that reflect specific wavelengths of light. Rather than using light-absorbing pigments, the color of photonic crystals is the result of their nanostructures.^1^ Of particular interest are stimuli-responsive photonic materials. These materials change their color based on specific stimuli such as light,^2−4^ temperature,^5−7^ and chemicals^8−10^ and may find applications in optical sensors, anti-counterfeiting labels, or heat-regulating window films.

A variety of both inorganic^6,11−16^ and organic^17−22^ photonic heat regulating window films have been reported previously. Cholesteric liquid crystals (CLCs) are an appealing class of responsive photonic organic materials for such windows.^23−25^ In the CLC phase, planarly aligned molecules are stacked to form a twisted helical structure that behaves similarly to a Bragg reflector. An advantage of such a CLC reflector is that it selectively reflects specific wavelengths of light while remaining transparent to the remainder. The exact wavelengths reflected by such a helix is determined by its pitch, the vertical distance across which the helix makes one full rotation.^26^ By choosing the appropriate liquid crystal mixture, it is possible to induce a phase transition from a non-reflective smectic state at lower temperatures to the cholesteric state at higher temperatures, creating a reflector that autonomously switches between transparent and reflective states.

This switch can be further improved by making use of the pre-transitional effect.^27^ This effect results in an extended temperature range across which the smectic state shifts into the cholesteric state. While such an extended shift can be achieved with certain mixtures of monomeric liquid crystals,^28^ their low viscosities restrict their usage as coatings. Oligomers by nature form more viscous mixtures, making them more suitable as scalable coating materials. When linear liquid crystal oligomer mixtures are used, long-range order is promoted. This can result in the stabilization or formation of the non-reflective smectic phase.^29^ The promotion of the smectic phase induces a more gradual phase transition from the smectic to cholesteric phase, tightening the helical twist over a wide temperature range and inducing a gradual blueshift of the reflected wavelength upon heating without affecting the visible region. A variety of cholesteric liquid crystal oligomers based on siloxanes^5,18^ and thiol-ene click chemistry^30,31^ have been synthesized to date. Owing to the large library of commercially available diacrylate liquid crystal monomers, oligomers with tunable functional and responsive properties can be designed.^17,31−37^ Making use of a hetero-oligomer allows for an additional degree of tunability and control over the phase behavior that is unavailable when only one liquid crystal monomer is used. However, structure property-relationships to program the thermochromic properties of these liquid crystal oligomers have been rarely reported.

Here, a systematic study is performed on a variable oligomer mixture using two common, commercially available liquid crystal monomers with identical core structures but differing by the length of their alkyl spacer. We use a variety of characterization methods to inspect the incorporation of both monomers into the oligomer. The underlying chemistry of such hetero-oligomers is of relevance to both liquid crystal oligomers, as presented in this work, and liquid crystal elastomers, which are created by the cross-linking of liquid crystal oligomers and are widely researched as actuators.^38−43^ By adjusting the ratio between the two monomers, a variety of short cholesteric liquid crystal oligomers are synthesized with reflection bands in the near-infrared region. The effects of the chemical composition on the room-temperature liquid crystal phase as well as the magnitude of the oligomers’ pre-transitional effect are studied, aiming for a reflection band at room temperature while maintaining the blueshift characteristic of the smectic–cholesteric transition. We show that the composition of a hetero-oligomer can be used to tune the reflective and thermochromic behavior of the material.

## Experimental Section

### Materials

Diacrylate mesogens 2-methyl-1,4-phenylene bis(4-((6-(acryloyloxy)hexyl)oxy)benzoate) (**C6M**) and (2-methyl-1,4-phenylene bis(4-(3-(acryloyloxy)propoxy)benzoate) (**C3M**) are purchased from Daken Chemical. ((3R,3aR,6S,6aR)-hexahydrofuro[3,2-*b*]furan-3,6-diylbis(4-((4-(((4-(acryloyloxy)butoxy)carbonyl)oxy)benzoyl)oxy)benzoate) (**4CD**) is purchased from BASF. 3,6-Dioxa-1,8-octane-dithiol (DODT), hexanethiol, and dipropylamine are purchased from Merck. Surfactant Byk-361N was purchased from BYK-Chemie. Dichloromethane (DCM), tetrahydrofuran (THF), and cyclopentanone are purchased from BioSolve. Stretched polyethylene terephthalate (PET) foil is purchased from Toyobo Film Solutions.

### Synthesis

Five different oligomers with varying ratios of **C6M** to **C3M** were synthesized. All solid compounds were added to a small brown glass vial. A small stirring bar was added to each vial. Quantities are chosen such that the molecular ratio of diacrylate:DODT is 2:1 for all mixtures. All mixtures contain a total of 2.5 wt % chiral monomer **4CD**. The remainder of the diacrylate is a varying mixture of **C6M** and **C3M**: the five mixtures 1–5 contain 0, 25, 50, 75, and 100 mol % **C3M** and the inverse fraction of **C6M**, respectively (Table 1). The total weight of material is approximately 1 g in each case.

**Table 1:** Experimentally Determined Properties of Synthesized Oligomers

oligomer	feed ratio, **C6M**:**C3M**	incorporated ratio, **C6M:C3M**	DP (based on NMR)	PDI (based on GPC)	*T*_g_ (°C)	*T*_S,Ch_ (°C)	*T*_Ch,I_ (°C)
**1**	100:0	100:0	2.3	1.93	–28	∼30–60	94
**2**	74:26	71:29	2.3	2.00	–26	<20–60	89
**3**	50:50	40:60	2.4	2.02	–24	N.A.	79
**4**	26:74	17:83	2.2	2.02	–21	N.A.	84
**5**	0:100	0:100	2.3	2.00	–18	N.A.	79

In a separate vial, the appropriate amount of DODT is added using a Finn pipet. The DODT is then dissolved in 3 mL of DCM and added to the reaction vial. An additional 2 mL of DCM is used to wash out the remainder of the DODT vial and added to the reaction vial. Ten microliters of dipropylamine is then added to the reaction mixture as a catalyst. The vials are shut tight with a lid and placed on a hot plate at 35 °C. The stirring is activated at 200 rpm, and the reaction is left overnight. Oligomers are dried by leaving them at 50 °C overnight to evaporate excess solvent.

A hexanethiol-end-capped oligomer mixture is synthesized by a similar procedure. The mixture contains 4 wt % **4CD** and a 3:1 ratio **C6M**:**C3M**. After synthesis of the oligomer following the procedure above, a 100% molecular excess of hexanethiol is added to the mixture. The vial is shut, and the mixture is again left to stir overnight at 35 °C. Oligomers are dried, and excess monothiol is evaporated by leaving the opened vial at 80 °C overnight.

### Characterization

^1^H-nuclear magnetic resonance (^1^H-NMR) samples are prepared from each mixture by pipetting 100 μL of the reaction mixture into a separate vial, evaporating the solvent at 50 °C for 24 h, and dissolving the dry material into 500 μL of deuterated chloroform. Spectra are measured using a Bruker Avance Core III 400 MHz spectrometer and analyzed using MestRenova. From the NMR spectra, the average oligomer length can be determined by eq 1, which is derived in the Supporting Information (Figure S1):
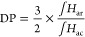
1where DP is the average chain length and ∫*H*_ar_ and ∫*H*_ac_ are the integrals corresponding to a set of aromatic hydrogens (8.15 ppm) and acrylate hydrogens (5.80–6.50 ppm), respectively.

Gel permeation chromatography (GPC) data is collected using a Shimadzu Prominence-i LC2030C 3D Liquid Chromatograph. After running the reaction for 12 h, GPC samples are prepared from each mixture by pipetting 30 μL of the reaction mixture into a separate vial and evaporating DCM at 50 °C for 24 h. The material is weighed, after which THF is added to create a stock solution of 1 mg/mL. One millimeter of this solution is passed through a 2 μm filter, and the measurement is performed.

Differential scanning calorimetry (DSC) measurements are performed with a TA Instruments Q2000. DSC is performed by exposing approximately 5 mg of every oligomer to a 5 °C/min temperature gradient. The samples are cycled between −50 and 150 °C three times, after which the final measurement is used to determine the glass transition temperature, *T*_g_, and the cholesteric-to-isotropic transition temperature, *T*_Ch,I_. The transition temperatures are rounded to the nearest integer.

### Coating Procedure

Coatings are produced by dissolving the oligomer mixtures into cyclopentanone (50 wt %). To this mixture was added 5 wt % (relative to the dry oligomer) of a 1 wt % surfactant solution in cyclopentanone. Stirring bars are added to the vials, after which they are closed with a lid and heated to 80 °C while stirring for 15–30 min until the viscous reaction product fully dissolves.

The inks are applied to the non-treated side of a 50 μm thick, 5 cm wide substrate via the wire bar coating technique using an RK K control coater. Both sides of a strip of PET are rinsed with isopropanol and then dried with compressed nitrogen. The top edge of a strip is taped to the top end of the bar-coater table. A wire bar with a 10 μm gap is placed in the holder and firmly pressed onto the strip. Three hundred and fifty microliters of the oligomer mixture is carefully applied at the wire bar/PET interface, ensuring that the whole width of the strip is covered. The machine is then turned on at a speed of approximately 1 cm/s. Resulting coatings were placed in the oven for 30 min at 60 °C to evaporate the remaining solvent. This process is repeated for all oligomers. Coatings produced from mixture 1 are further referred to as coating **1**, and so on. After evaporation of cyclopentanone, the coatings are approximately 5 μm thick.

### Temperature-Dependent Transmission Experiments

All UV–vis data was collected within 24 h of production of the coating in question unless specified otherwise. Transmission spectra are measured on a small strip cut from the coated foils with air as a baseline. The baseline is taken with the heater module (Linkam LNP-96S) mounted in the machine (PerkinElmer LAMBDA 750 UV/vis/NIR spectrophotometer). After the baseline is taken, a small strip is cut from the coated foil and clamped onto the heating module. The stage is set to 20 °C, and a measurement is taken. After completion, the coating is heated by 10 °C at a rate of 5 °C/min and then left to rest at that temperature for 2 min. The next measurement is then taken, and this cycle is repeated until the measurement no longer shows a reflection band. Once this happens, the hot stage is set to cool in intervals of 10 °C, following the same procedure as before until cooled down to 10 °C. The strip is then removed. In cases where the data is extremely noisy due to machine fatigue from extended use, the data is smoothed by using the Origin software function “adjacent averaging” setting on 30.

## Results

### Synthesis and Characterization of the Liquid Crystal Hetero-Oligomers

**Figure 1:**
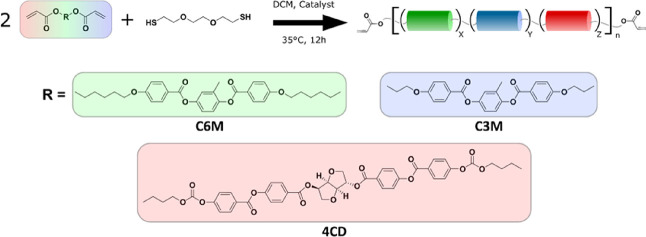
Schematic representation of the chemicals used to perform the oligomerization reaction. X, Y, and Z vary based on the reaction feed.

For the fabrication of the near-infrared (NIR) reflectors, thermochromic liquid crystal hetero-oligomers are synthesized with varying ratios of **C6M** and **C3M** to form five different oligomers (Table 1). Figure 1 schematically depicts the reaction procedure as well as the chemical structures of the compounds used. The oligomers are obtained by a thiol-ene reaction between a dithiol, the diacrylate monomers **C6M** and **C3M,** and 2.5 wt % of chiral dopant **4CD** to induce a cholesteric phase. With an assumed refractive index of 1.6^44^ and an HTP of 55 μm^–1^, a NIR-reflected wavelength of 1164 nm can be expected.^26^ The synthesized oligomers were designed to have an average length of 2 monomer units, controlled through the reaction feed, where using twice as much acrylate as dithiol should result in the desired length. Although the average result is the formation of dimers, both free monomers and longer oligomers will also be part of the final mixture. Short hetero-oligomers were selected with processability and thermochromic response in mind: one-component monomeric mixtures show no pre-transitional effect,^17^ while long oligomers are difficult to align during coating and exhibit slow or no responses due to their relatively high viscosity.

The resulting oligomers are characterized with ^1^H-NMR, GPC, and DSC (Figures S2–S8). ^1^H-NMR spectra show that both **C3M** and **C6M** are present in expected amounts. Peaks corresponding to unreacted dithiol chain extenders are not observed in any of the spectra, indicating that the reaction has gone to completion. Although **4CD** is present in only small quantities, peaks corresponding to its isosorbide core can be seen in all spectra. The average oligomer length of each mixture can be calculated using the relative integrals of the remaining acrylate groups and the aromatic hydrogens of the liquid crystal units (eq 1 and Figure S2)*.* The resulting oligomer lengths are in reasonable agreement with the designed oligomer length of 2 units, with small variations between the various oligomer mixtures (Table 1). The reaction can thus be assumed to have gone to completion.

The GPC profiles of all synthesized oligomers show four distinct major peaks followed by a smooth tail. Since all oligomers contain only small amounts of **4CD**, this monomer does not significantly affect the profiles. As the oligomers synthesized have an average DP of 2, it is possible to distinguish peaks of individual oligomer lengths. The rightmost peaks in oligomers **1** and **5** are experimentally shown to belong to monomeric **C6M** and **C3M**, respectively (Figure S5). Subsequent peaks show a regular increase in mass. Although this mass increase is calibrated toward polystyrene rather than the synthesized oligomers, it is likely that the three peaks following the monomers correspond to the dimers, trimers, and tetramers. The remaining oligomer lengths are captured by the broad tail as the relative differences between subsequent chain lengths become too small to show distinguishable individual peaks. For all major peaks, those corresponding to oligomer **1** (**C6M** only) lie to the left of the corresponding peaks of oligomer **5** (**C3M** only) as expected. Oligomer **3**’s peaks consistently appear between those of **1** and **5**, with its peaks slightly broadened due to a variety of species with the same DP existing within this mixture. By deconvoluting the GPC data and integrating the peaks, it is found that approximately 25 wt % of the material has a DP of 2 (Figure S6 and Table S1*)*.

**Figure 2:**
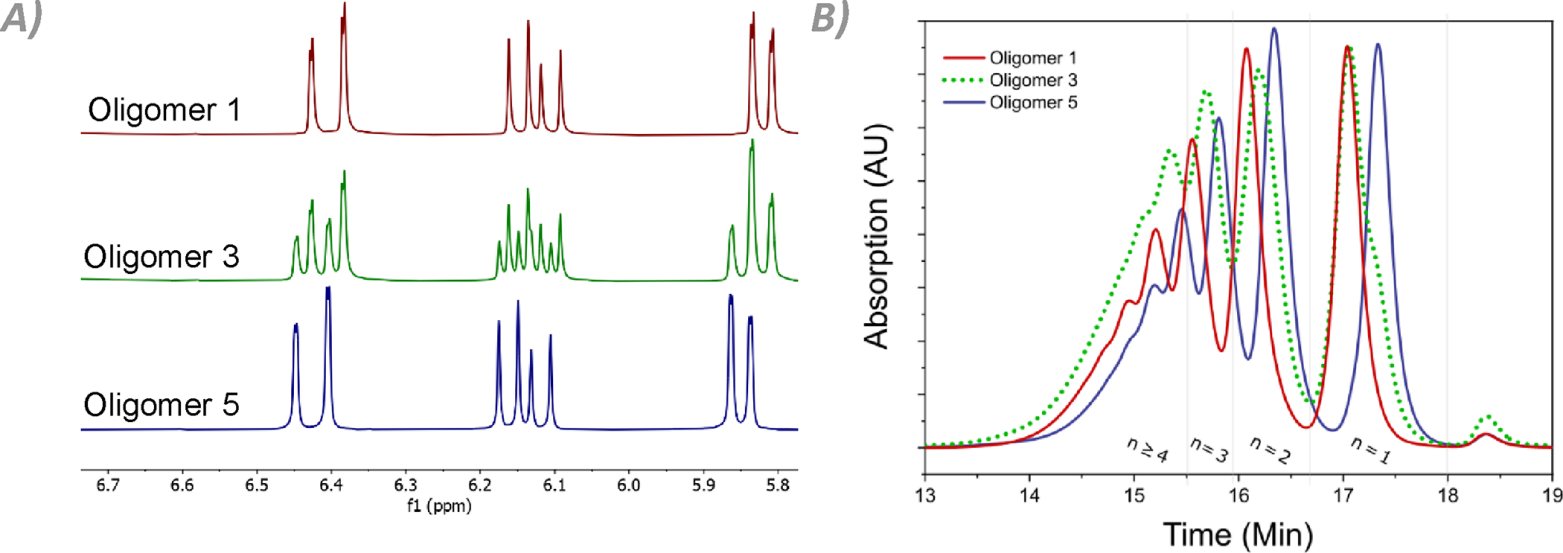
Measurements demonstrating the inequal reactivities of C6M and C3M. (A) Part of the ^1^H-NMR spectrum showing the remaining acrylate groups in oligomers 1, 3, and 5. (B) GPC measurements for oligomers 1, 3, and 5, normalized to the monomer peaks.

When comparing the ^1^H-NMR and GPC data of the different oligomers, they provide evidence that **C6M** and **C3M** have different reaction rates with the dithiol spacer. Upon completion of the reaction, oligomers containing both monomers show that **C3M** has reacted substantially more than would be expected in the case of equal reactivity of both monomers. This is particularly evident when the NMR signals of the remaining acrylate group in oligomer **3** are considered (Figure 2A), where equal amounts of both monomers were used. The small positional variations between the acrylate NMR signals of both original monomers (approximately 0.02 ppm) allow for a clear distinction between the different monomers when both are incorporated into the oligomer. Using the NMR spectra of oligomers **1** and **5** as references, it can be clearly seen that the signals corresponding to **C3M**’s moieties are significantly less prominent than those of **C6M** in oligomer **3**. This difference is quantified by integrating the peaks of the two different acrylates individually and determining their ratio. This ratio is then converted to determine the actual composition of the oligomer chains (Table 1). In all cases, the incorporated ratio of the two monomers does not correspond to the feed ratio but shows an increase of **C3M** compared to **C6M**. This difference remains observable on repeats of the synthesis and can only be explained if **C3M** reacted more with the chain extender than **C6M**.

NMR signals corresponding to the alkyl tails of both monomers formed during the reaction also indicate a higher rate of reaction for **C3M** and **C6M** in all oligomers (Figures S9 and S10). By modeling the reactions of both monomers with the dithiol spacer as two second-order reactions and using the initial and final ratios of the acrylate groups, it can be determined the rate constant of **C3M**’s reaction with the dithiol spacer is approximately twice that of **C6M**, which is a significant difference (Figure S11).

The GPC data is in agreement with this conclusion (Figure 2B). When considering the monomer peaks in oligomer **3**, the quantity of free **C6M** after reaction completion is far greater than the quantity of free **C3M**. This finding provides another indication that **C6M** is incorporated into the oligomer chain at a lower rate than **C3M**. GPC data for the remaining oligomers follows a similar pattern and is provided in the Supporting Information (Figure S7). Different reaction rates of acrylates with thiols have been reported previously,^45^ but to our knowledge, this has not previously been shown in liquid crystal oligomers. Molecular weight may have some impact on this, but the weights of the two monomers used in this work differ by only about 10 percent. The higher reactivity of the acrylate groups of **C3M** compared to those of **C6M** might instead be explained by the fact that the electron-withdrawing effect of the aromatic core more strongly affects the reactive site of **C3M**, making the acrylate group more electrophilic toward the thio-compound. This is also apparent from the ^1^H-NMR spectra, where the signal of the methylene group connected to the acrylate moiety is found at lower field for **C3M** than for **C6M**, indicating a higher inducing effect. Diffusion-ordered spectroscopy (DOSY)-NMR on oligomer **3** shows that **C3M** is present more than **C6M** in longer oligomers (Figure S12). As oligomer **3** was synthesized using equimolar amounts of **C3M** and **C6M**, this can only be explained by **C3M** being more likely to react than **C6M**.

The transition temperatures determined from DSC data (Table 1 and Figure S8) follow a clear pattern, with an increasing glass transition temperature and decreasing isotropic transition temperature as the **C3M** content increases. The only outlier is the isotropic transition temperature of oligomer **3**, which might be due to the slightly higher DP compared to the other oligomers. The smectic–cholesteric transition does not appear in the DSC traces, most likely as a result of the pre-transitional effect broadening the temperature range of this transition to such an extent that no specific peaks can be observed. POM imaging of the coatings, however, provides some insight regarding this transition for oligomers **1** and **2**, where the pre-transitional effect is more pronounced (Table S4). This broad temperature range for the transition of these two oligomers is included in Table 1.

### Thermochromic NIR-Reflective Coatings

**Figure 3:**
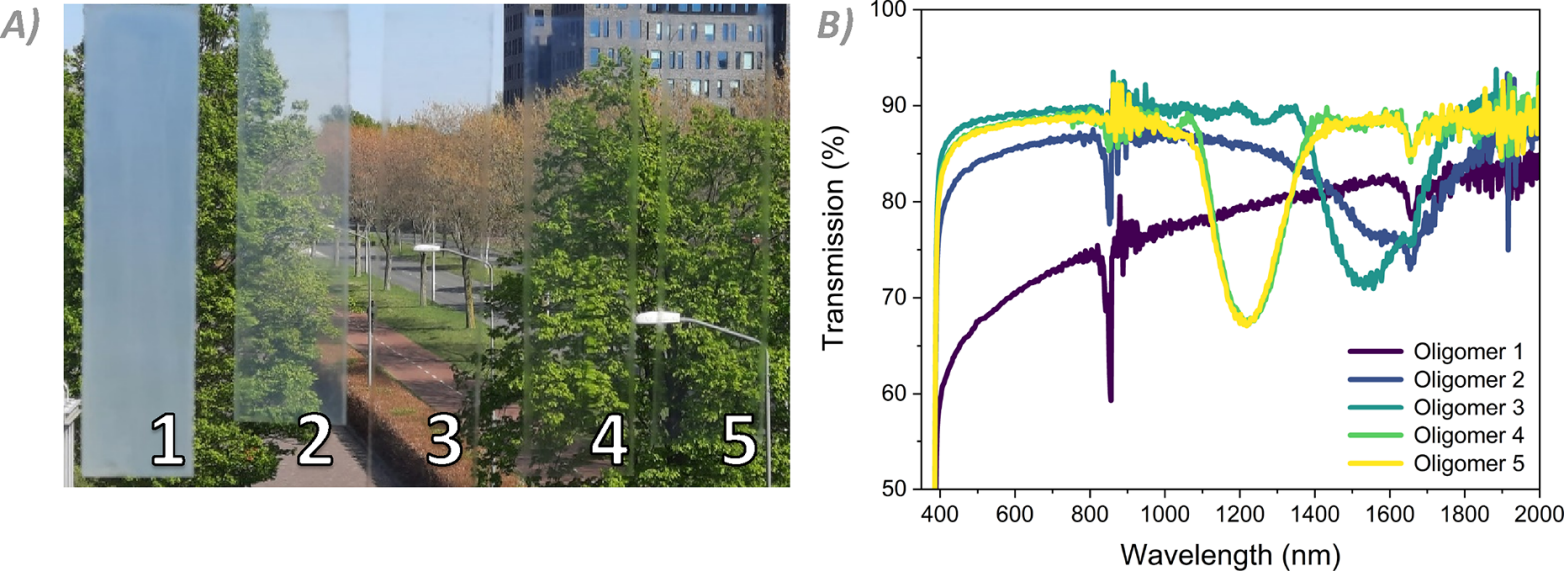
Room temperature behavior of all coatings after 2 weeks at rest. (A) Coatings of oligomer **1** through **5**, with increasing C3M content from left to right. (B) UV–vis spectra of the same coatings at room temperature. The sharp peaks visible at 890 nm are a result of the measurement procedure rather than defects in the samples.

Solutions of oligomer mixtures **1** to **5** in cyclopentanone were bar-coated onto PET films to investigate their reflective properties. Immediately after evaporation of the solvent, the coatings are optically transparent. However, after leaving the coatings at room temperature for 2 days, coating **1** becomes opaque as a result of scattering (Figure 3A). Coating **2** turns slightly scattering, while coatings **3**, **4**, and **5** remain transparent over the same timeframe. The strong visual distinction between the coatings can be attributed to a difference in mesophase at RT. Transmission spectra show that coating **1** does not possess a reflection band at RT, instead forming a scattering phase (Figure 3B). This lack of a reflection band is likely the result of a multi-domain smectic phase. All other coatings show reflection bands, implying that they possess a cholesteric phase at RT.

The calculated theoretical wavelength of 1164 nm is in good approximation to the reflection bands exhibited by coatings **4** and **5**, but coatings **2** and **3** have their reflection band centered around 1600 nm. In the latter case, the coatings have not fully completed their transition to the cholesteric phase at room temperature due to the pre-transitional effect. They are essentially in between the cholesteric and smectic phases, making their reflection bands shift toward wavelengths that are longer than theoretically expected. The increased **C3M** content promotes the formation of the cholesteric phase at RT. As a result, coatings **2** through **5** show increasing cholesteric character compared to coating **1**, which slowly stabilizes into its smectic state.

**Figure 4:**
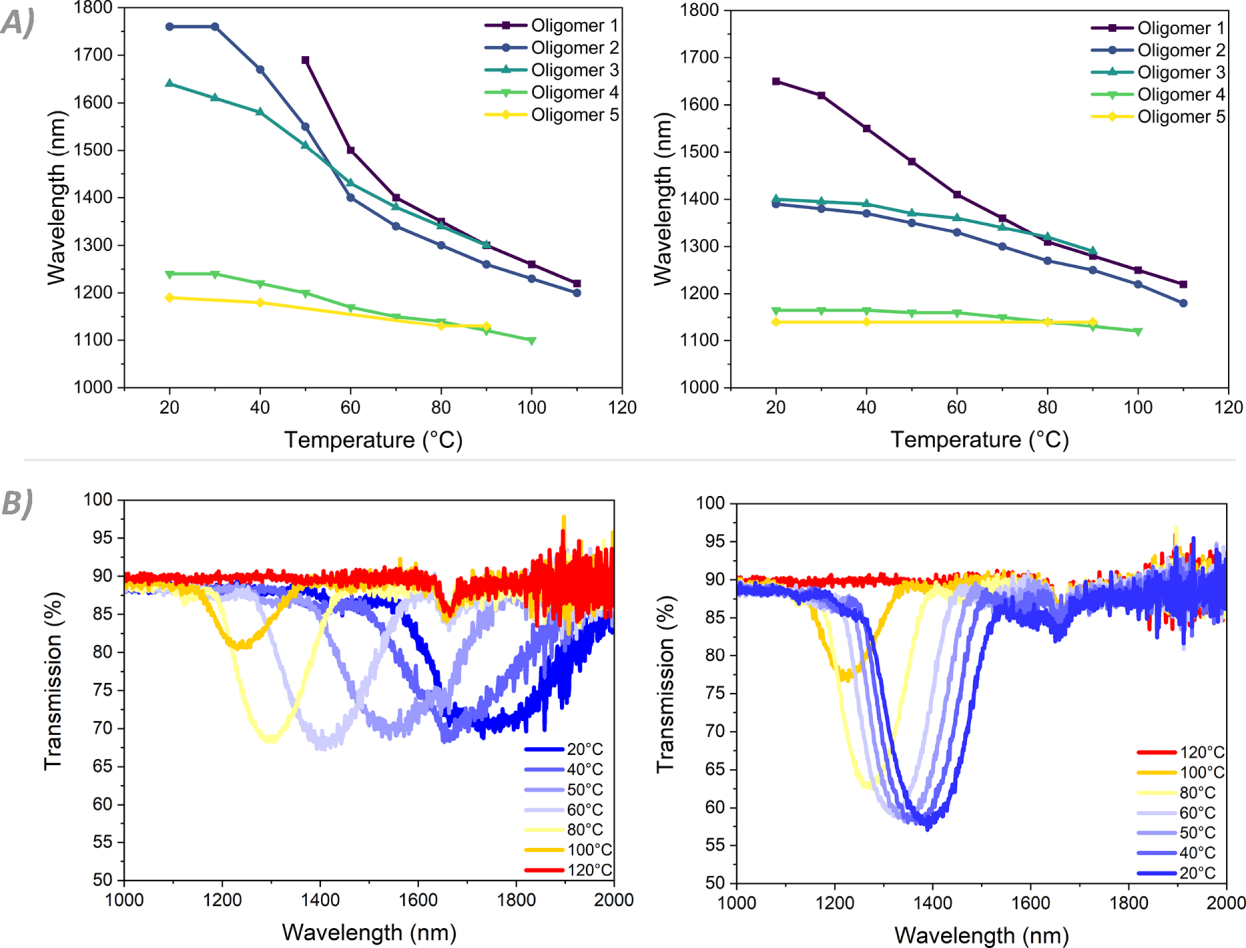
Temperature response of the oligomer coatings. (A) Peak reflective wavelengths of the coatings during the heating (left) and cooling (right) cycles. (B) Full response for oligomer **2** during the heating (left) and cooling (right) cycles.

To determine the thermochromic response of all coatings, temperature-dependent optical measurements were performed. During these experiments, coatings are heated or cooled at a rate of 5 °C per min and then left at the designated temperature for 2 min before the measurement is performed. The initial and final peak reflected wavelengths, as well as the difference between them, are noted in Table 2 for all coatings. The complete measurements for all coatings are provided in the Supporting Information (Figure S13). When the thermal response of all coatings is examined side-by-side, a clear pattern emerges (Figure 4A). Coating **1**, as previously noted, possesses no reflection band at RT. However, upon heating the coating, an initially broad reflection band centered around 1700 nm becomes visible starting at 50 °C. The coating then blueshifts rather steeply due to the pre-transitional effect, which flattens off toward 1200 nm as the coating is further heated until the non-reflective isotropic state is entered at 120 °C. Conversely, coating **5** exhibits a well-defined reflection band at RT. Upon heating, it shows a much smaller shift of the reflected wavelength of approximately 60 nm. This absence of a notable pre-transitional effect indicates that oligomers prepared from **C3M** are well above the smectic–cholesteric phase transition at RT, if they possess a smectic phase at all. Coating **5** remains fully transparent and cholesteric for months after it is produced, lending further credence to the assumption that it is stable in its cholesteric state at RT.

**Table 2:** Measured Peak Reflected Wavelengths of the Synthesized Oligomers

oligomer	RT peak reflected wavelength (nm)	final peak reflected wavelength (nm)	total reflection band shift (nm)
**1**	none	1260	400
**2**	1750	1240	510
**3**	1610	1300	310
**4**	1240	1110	130
**5**	1190	1130	60

The combination of the two monomers leads to a combination of the properties exhibited in coatings **1** and **5**. As in the homo-oligomers, the **C3M**-containing oligomers promote the cholesteric phase as opposed to the smectic behavior of **C6M**. As a result, adding **C3M** to the **C6M** homo-oligomer induces a room-temperature cholesteric phase. While changes in the reflection band do occur at lower temperatures, the most significant change is always around 40 to 60 °C, where the pre-transitional effect of the **C6M**-containing oligomers is strongest. As more **C3M** is added to the mixture, the reflection band becomes narrower at RT and the isotropic temperature at which no reflection is measured decreases, as does the magnitude of the pre-transitional effect and, thus, the blueshift. These changes are all gradual, so it is possible to tune the composition toward desired properties by systematically adjusting the ratio of the two monomers.

The greatest reflection band shift of 510 nm occurs in coating **2** (Figure 4B and Table 2). This shift appears greater than that of coating **1**—it seems likely, however, that part of the shift of coating **1** remains obscured due to its slow transition, making the true shift larger. The shift decreases further for coating **3** and **4** as the composition shifts toward coating **5** and the cholesteric phase becomes more and more dominant at RT, weakening the pre-transitional effect. After the coatings are heated up to approximately 100 °C to fully pass into their cholesteric phase, all coatings achieve approximately the same peak reflected wavelength of 1200 nm, with small variations due to exact compositions and chain lengths.

**Figure 5:**
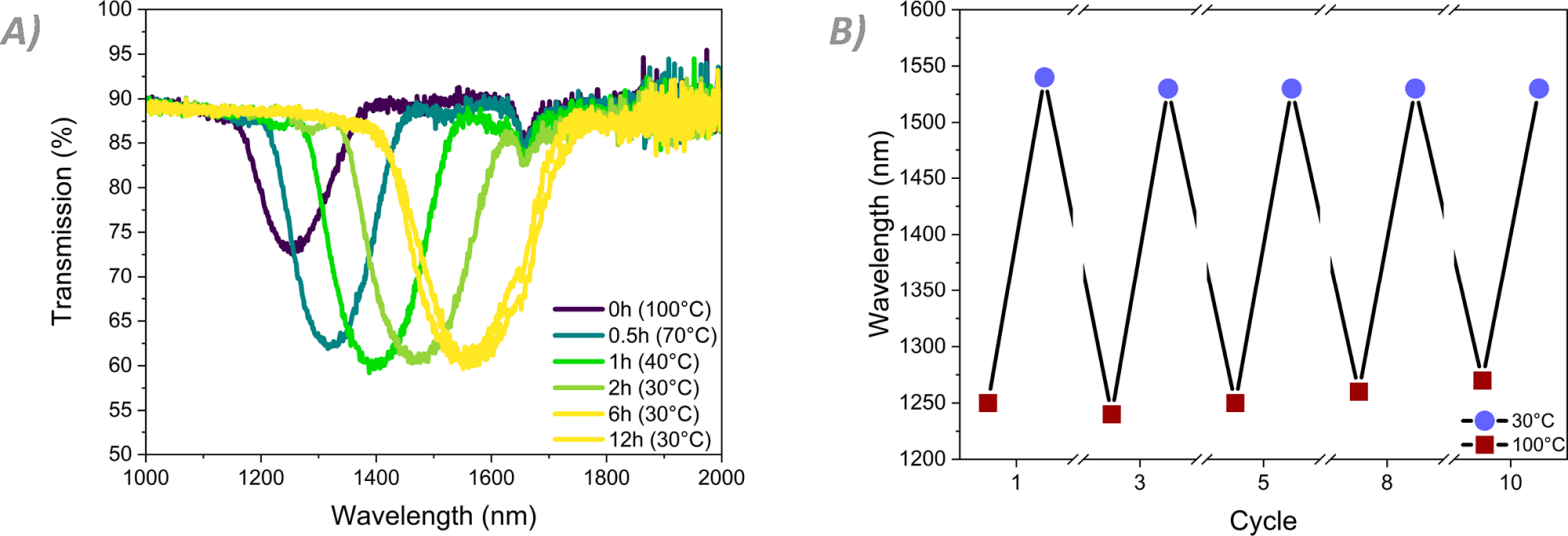
Spectra showing the reflective behavior of coating **2** upon cooling from the isotropic phase. (A) Reflection bands observed during the first cooling cycle, showing slow but satisfactory recovery of the reflection band and its position. (B) Cyclic position of the peak reflected wavelength at 100 °C after cooling from the isotropic state and 30 °C after 6 h at rest.

When the coatings are cooled from their isotropic state, they recover their cholesteric alignment within seconds. However, as the coating is cooled to room temperature, the shift of the reflected wavelength starts to lag when compared to equivalent temperatures during the heating cycle. A possible explanation for this delay is the relatively high viscosity of the oligomers. During heating, the viscosity continuously decreases, making the rearrangement of the molecular alignment easier. During cooling, the opposite is true, leaving the coating kinetically trapped as the cooling rate outpaces the molecular rearrangement. However, since a low DP mixture is used, the viscosity is not high enough to completely prevent the molecular alignment from rearranging. If the coating is cooled to 30 °C at a rate of 1 °C/min and left overnight, then after approximately 6 h, the coating recovers to its initial reflection wavelength (Figure 5A). This cycle of heating and cooling was performed 10 times on the same strip of coating **2**. The position of the reflection band at the start and end of the cooling cycles remained virtually identical for all measurements, returning to within 5% of the peak reflected wavelength of the first cycle (Figure 5B).

### Adjustability of the Hetero-Oligomer Composition

**Figure 6:**
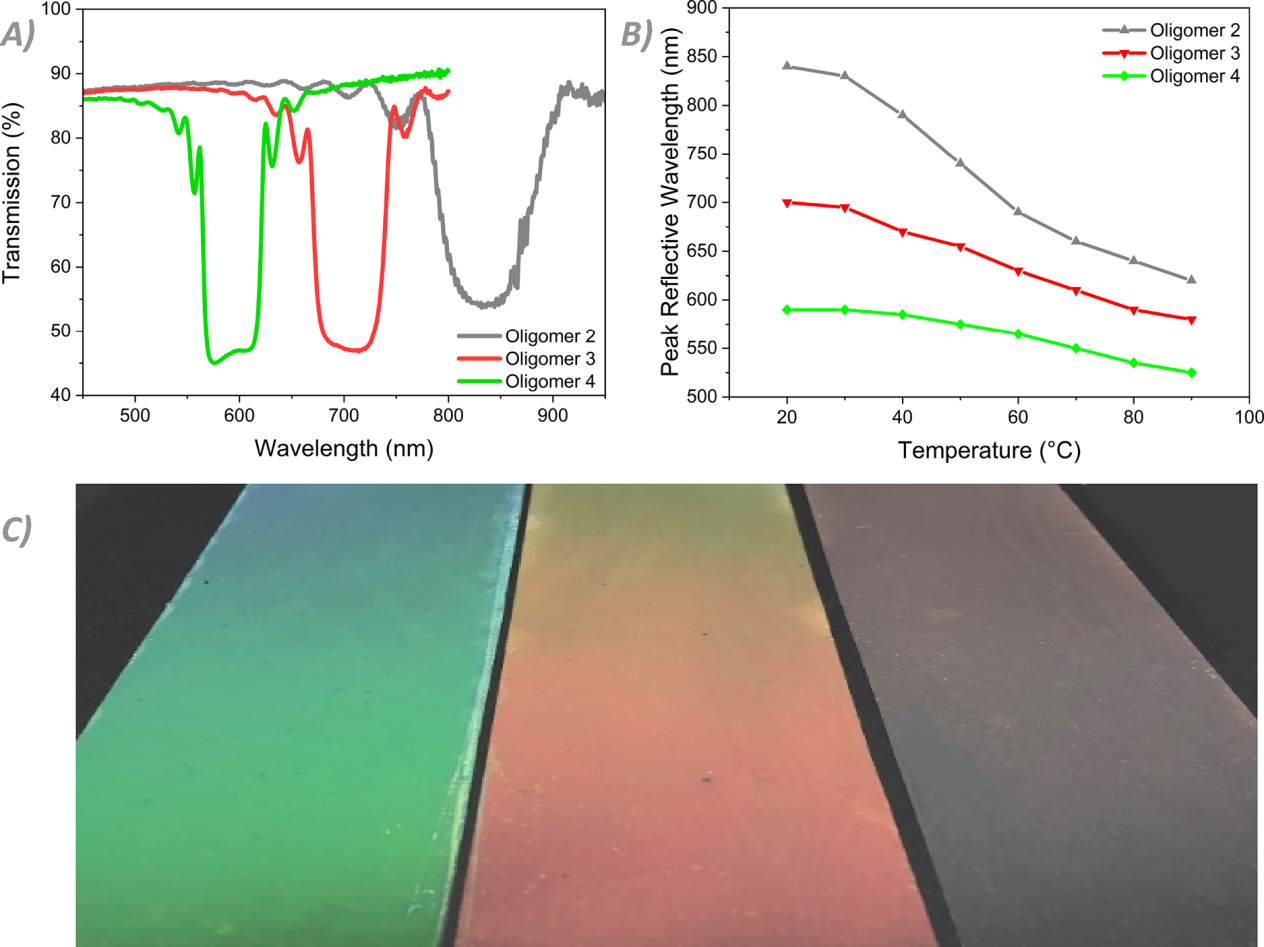
(A) Transmission spectra of oligomers **2**, **3**, and **4** with 5.5 wt % **4CD** at RT. (B) Peak reflective wavelengths of the modified coatings upon increasing the temperature. (C) Picture of the oligomer coatings at RT, demonstrating the large differences in the reflected wavelength.

Molecular adjustments of the hetero-oligomers were made to show that the reflection wavelength can be tuned and that the oligomers can be easily end-capped. The incorporation of a larger quantity of chiral dopant—5.5 wt % as opposed to the 2.5 wt % in the original mixture—shifts the reflection band toward the visible spectrum (Figure 6A). As the position of the reflection band is inversely proportional to the amount of chiral dopant,^26^ this increase of the chiral dopant concentration results in approximately halving the peak reflected wavelengths at room temperature. This matches with our findings (Figure 6A). The optical properties of the coatings are comparable to those of the NIR-reflective coatings. Oligomer **2**, with more **C6M**, has a room temperature reflection peak at a longer wavelength than oligomers **3** and **4**. The large visual difference (Figure 6C), as seen in the previous set of coatings, is purely an effect of the different ratios of the two monomers. Furthermore, the intensity of the pre-transitional effect follows the same trend, with the total shift of the peak reflected wavelength decreasing as the **C3M** content increases (Figure 6B*)*. Cholesteric oligomers can thus be designed to have specific RT reflection bands and wavelength shifts by adjusting the monomer ratio in addition to the concentration of **4CD**. The chiral dopant concentration can be used to tune the reflected wavelength achieved upon formation of the cholesteric phase, while the ratio of **C6M** to **C3M** can be used to control the extent of the pre-transitional effect and thus affect the reflected wavelength at room temperature.

**Figure 7:**
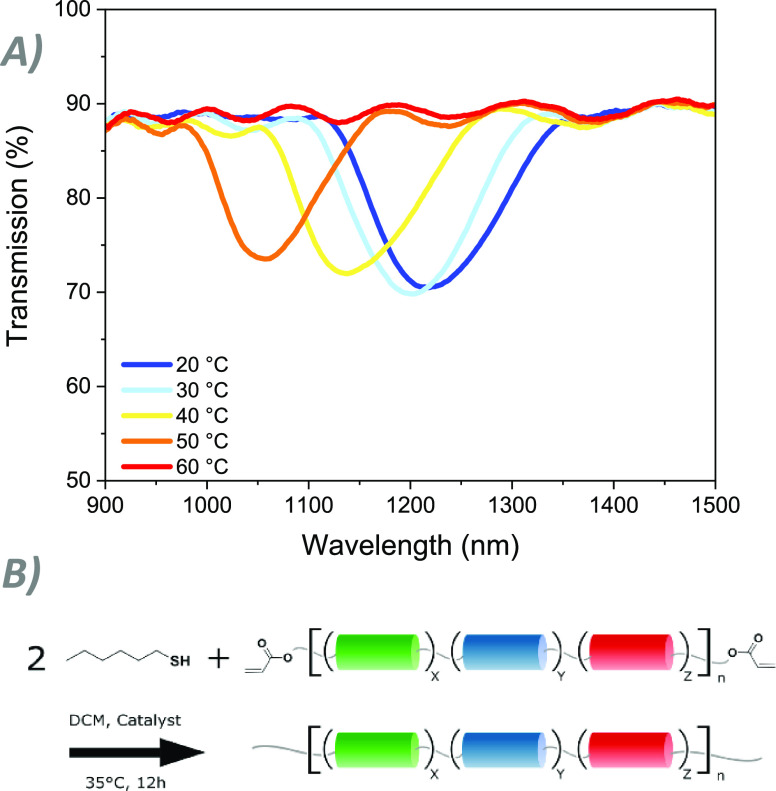
(A) Temperature response of the reflection band of the thiol-modified oligomer **2**. (B) Modified reaction scheme following the same procedure as the original synthesis.

As a final adjustment, an oligomer was produced with its functional acrylate groups reacted with hexanethiol following the same thiol-ene procedure outlined for the original synthesis (Figure 7). For this example, a simple aliphatic tail was used to end-cap the oligomer, which may improve the long-term stability of the oligomers by removing the reactive acrylate group. However, the high versatility of the thiol–Michael addition reaction allows us to functionalize the oligomers with a large library of thiol-containing compounds. The composition of the initial reaction mixture was modified such that after reaction with the monothiol, the total chiral dopant concentration would be 3.5 wt %. The resulting oligomer mixture was subsequently characterized by NMR, GPC, and DSC (Figure S14), which indicate a conversion of >99%. The coating produced from this modified oligomer maintains an RT reflection band as well as a clear pre-transitional effect even with the addition of aliphatic carbon tails. When compared to the untreated oligomers, the temperature response is initially similar. However, the modified oligomer does not achieve the same total shift of the reflection band as it enters the isotropic state at a lower temperature. This may be a result of a larger portion of the molecule consisting of aliphatic tails. Despite this difference, the experiment demonstrates that the oligomers can be further functionalized and programmed while maintaining thermochromic properties.

## Conclusions

In this work, we successfully synthesized liquid crystal oligomers to produce thermochromic NIR-reflective cholesteric coatings. The thermochromic behavior of the oligomer coatings can be tuned by varying the ratio of monomers used during synthesis. However, one must consider the fact that the kinetics of the oligomer synthesis strongly depend on the selected monomers and that the ratio at which the different monomers are incorporated into the main chain oligomer varies as a result. These effects can be clearly observed by both ^1^H-NMR and GPC measurements. While this does not adversely affect the properties of the coatings produced from these mixtures in this work, it means that the overall distribution of the monomers across oligomer lengths is not proportional to the molecular feed. This could significantly affect material properties depending on the chosen monomers. For example, when long oligomers or polymers are formed, the observed difference of the reaction rates could result in block copolymers rather than random copolymers. Additionally, the chiral dopant **4CD** may have a rate of reaction different from the monomers, resulting in it potentially being incorporated to a greater or lesser extent than expected.

It was demonstrated that a cholesteric homo-oligomer bearing a strong pre-transitional effect can be modified to fabricate thermochromic coatings with an NIR reflection band at room temperature by incorporating a second monomer into the chain. We have further demonstrated that the reflective wavelength of these coatings could be predictably adjusted by altering the chiral dopant concentration and the ratio of the non-chiral monomers and that the reactive end-groups can be end-capped while maintaining thermochromic properties. This could be exploited in the future to prevent long-term polymerization of the acrylate groups, add additional functional groups to the oligomers, and tune properties such as the transition temperature and reflection band shift. As the oligomers are produced following a simple one-pot synthesis and applied as a coating, this system is potentially scalable for applications such as thermochromic NIR-reflecting window foils.

## Supporting Information Available

^1^H-NMR spectra, GPC profiles, DSC curves, UV–vis spectra, POM images, and modeling of reaction kinetics (PDF)
