# Supplementary material for: Programming Thermochromic Liquid Crystal Hetero-Oligomers for Near-Infrared Reflectors: Unequal Incorporation of Similar Reactive Mesogens in Thiol-ene Oligomers
Source: Macromolecules. 2022 Dec 27;56(1):59–68. doi: 10.1021/acs.macromol.2c02041 (PMC9835980; doi:10.1021/acs.macromol.2c02041)
Supplement: Supplementary file 1 — ma2c02041_si_001.pdf [file ma2c02041_si_001.pdf]

Supporting Information

Programming Thermochromic Liquid Crystal Hetero-Oligomers for Near-Infrared Reflectors: Unequal Incorporation of Similar Reactive Mesogens in Thiol-ene Oligomers

Henk Sentjens,[1,2] Augustinus J.J. Kragt,[1,3,4] Johan Lub,[1] Mart D.T. Claessen,[1] Vera E. Buurman,[1] Joris Schreppers,[1] Henk A. Gongriep,[1] Albert P.H.J. Schenning[1,2,*]

[1] Laboratory of Stimuli-Responsive Functional Materials and Devices (SFD), Department of Chemical Engineering and Chemistry, Eindhoven University of Technology (TU/e), P.O. box 513, 5600 MB Eindhoven, The Netherlands. E-mail: a.p.h.j.schenning@tue.nl

[2] Institute for Complex Molecular Systems, Eindhoven University of Technology (TU/e), P.O. Box 513, 5600 MB Eindhoven, The Netherlands

[3] Faculty of Architecture, Delft University of Technology, Julianalaan 134, 2628 BL, Delft,

[4] ClimAd Technology, Valkenaerhof 68, 6538 TE, Nijmegen, The Netherlands

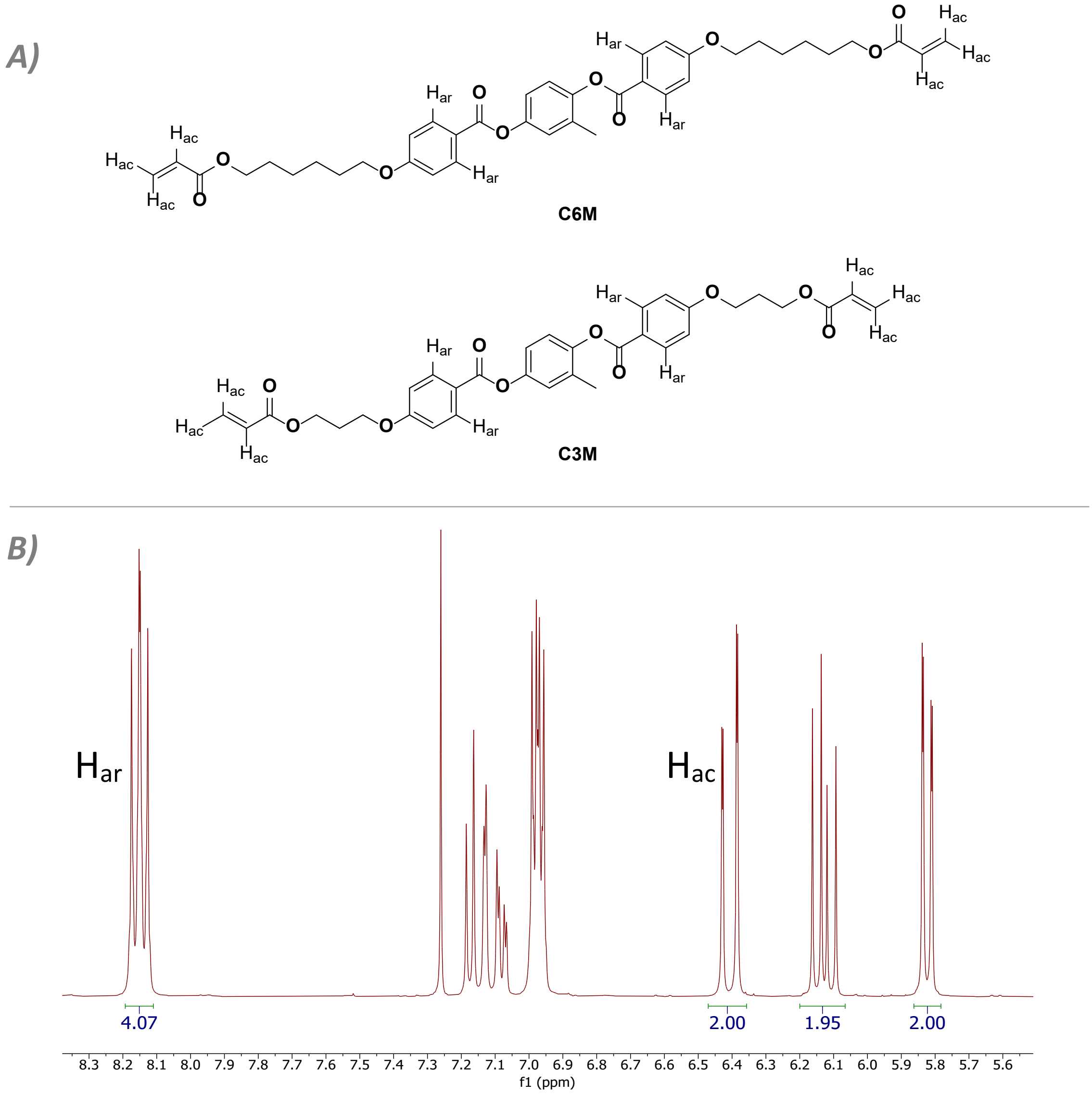

Figure S.1: Structures used to derive DP. A) Chemical structures of monomers ***C6M*** *and* ***C3M****.*
B) Relevant portion of the ^{1}H-NMR spectrum with indicated signals used for the calculation.

To determine the chain length of the oligomers, the ratio of the reactive acrylate hydrogens H_{ac} (5.75-6.45ppm) and the aromatic hydrogens H_{ar} (8.15ppm) was calculated. Assuming the acrylates only react when they are involved in a chain-extension, the expected ratio H_{ar}/H_{ac} can be written as

H_{ar}/H_{ac} = 2/3*DP

Where DP is the chain length. Therefore $\mathrm{DP} = \frac{3}{2} * \frac{\int H_{ar}}{\int H_{ac}}$

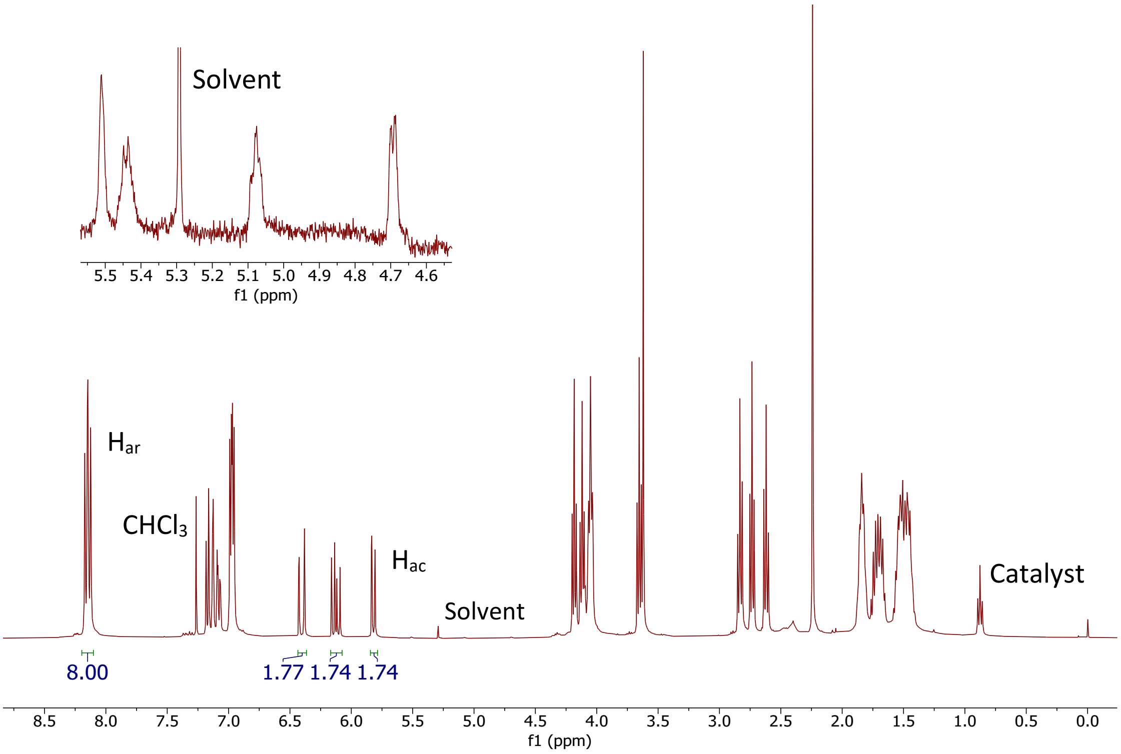

Oligomer 1

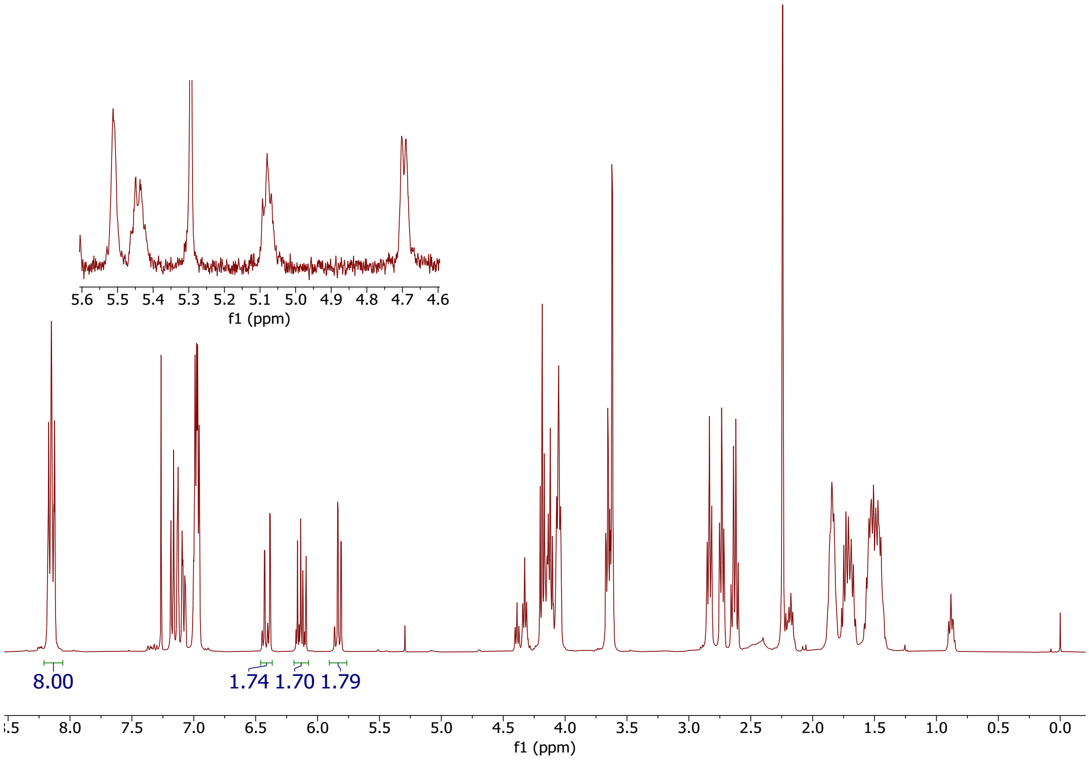

Oligomer 2

Figure S.2: ^{1}H-NMR Spectra of synthesized oligomers ***1*** *and* ***2****. Peaks indicated in oligomer 1 are equivalent in all other spectra. The top-left insets shows peaks corresponding to the isosorbide core of the chiral dopant along with the solvent peak. The indicated signals H_{ac} and H_{ar} correspond to the hydrogens in the oligomer equivalent to the indicated hydrogens in the monomer.*

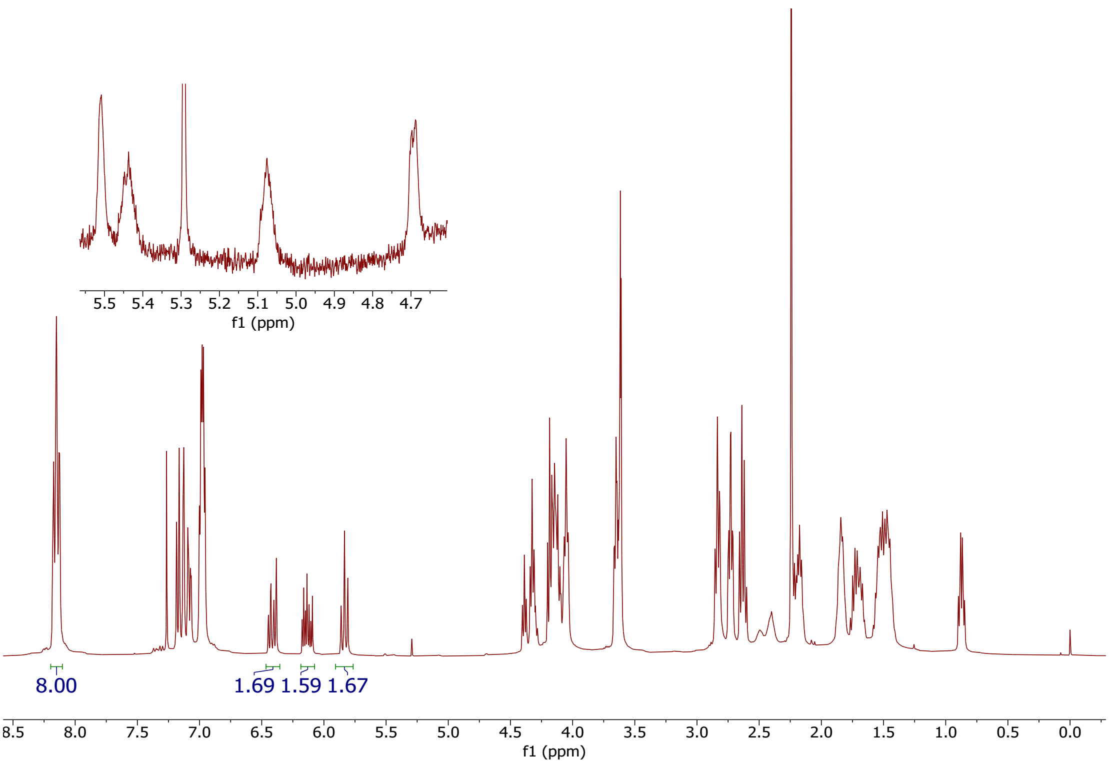

Oligomer 3

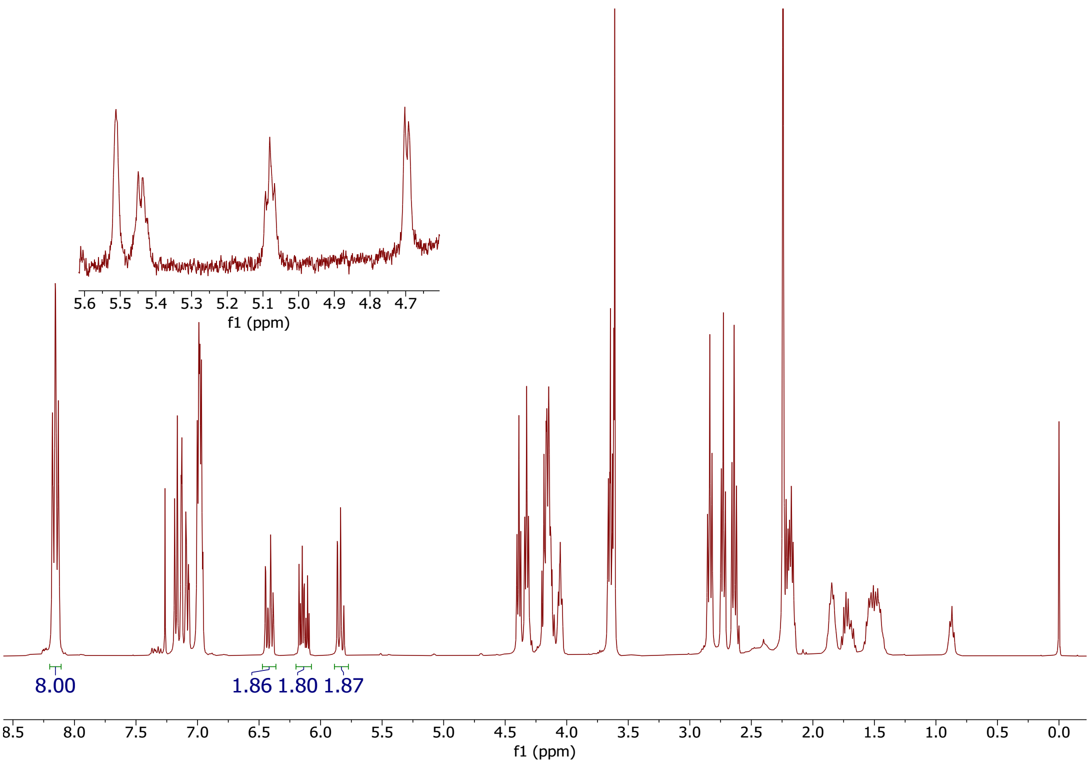

Oligomer 4

*Figure S.3: ^{1}H-NMR Spectra of synthesized oligomers **3** and **4***

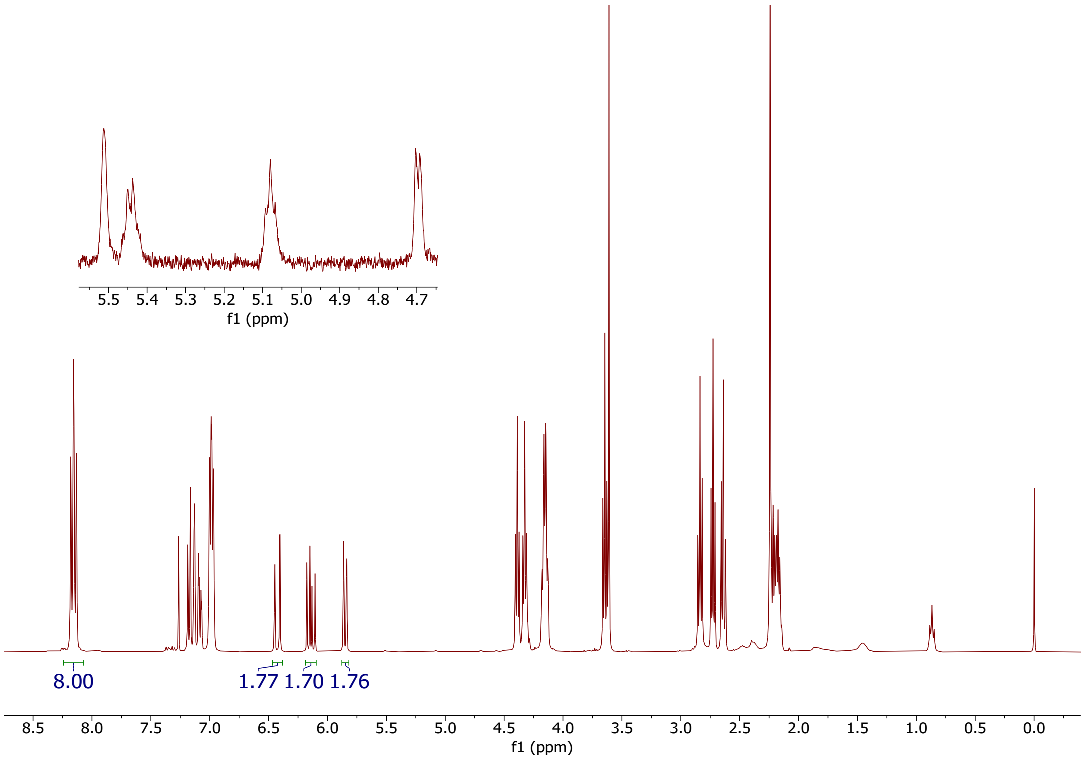

Oligomer 5

*Figure S.4: ^{1}H-NMR Spectra of synthesized oligomers **5***

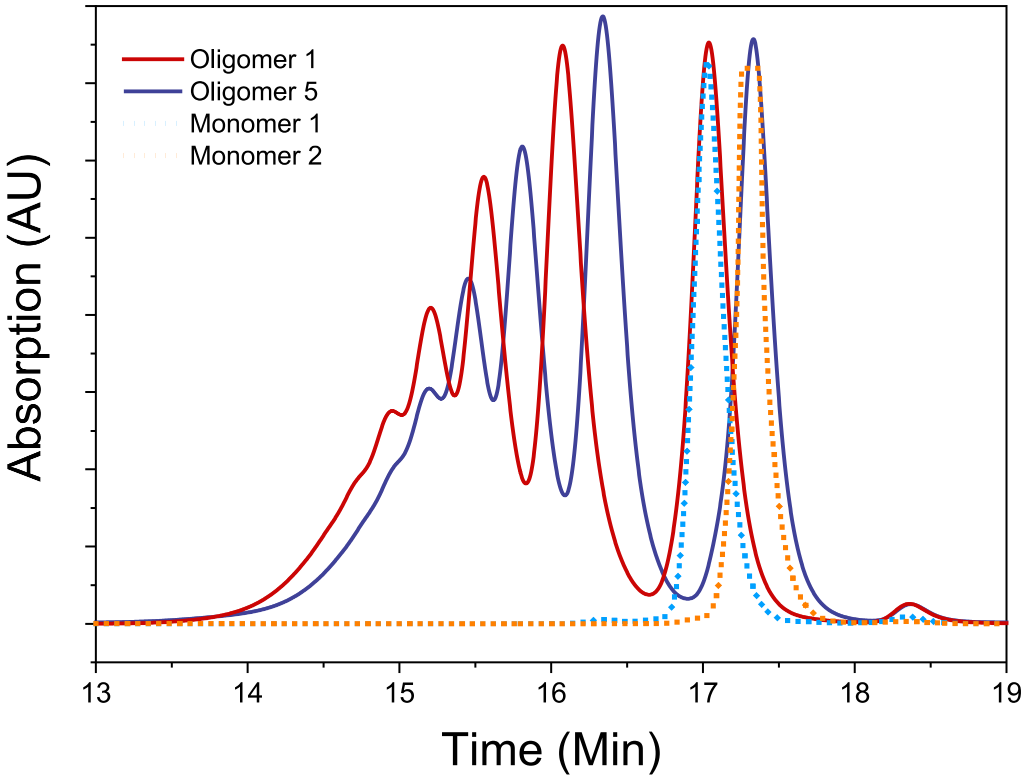

*Figure S.5: GPC profiles of oligomers **1** and **5** and their constituent monomers. The peak at 18.4 minutes originates from a stabilizer in the solvent used for the experiments. The areas under the peaks are integrated and compared to an internal standard by the software to determine the PDI reported in the main text.*

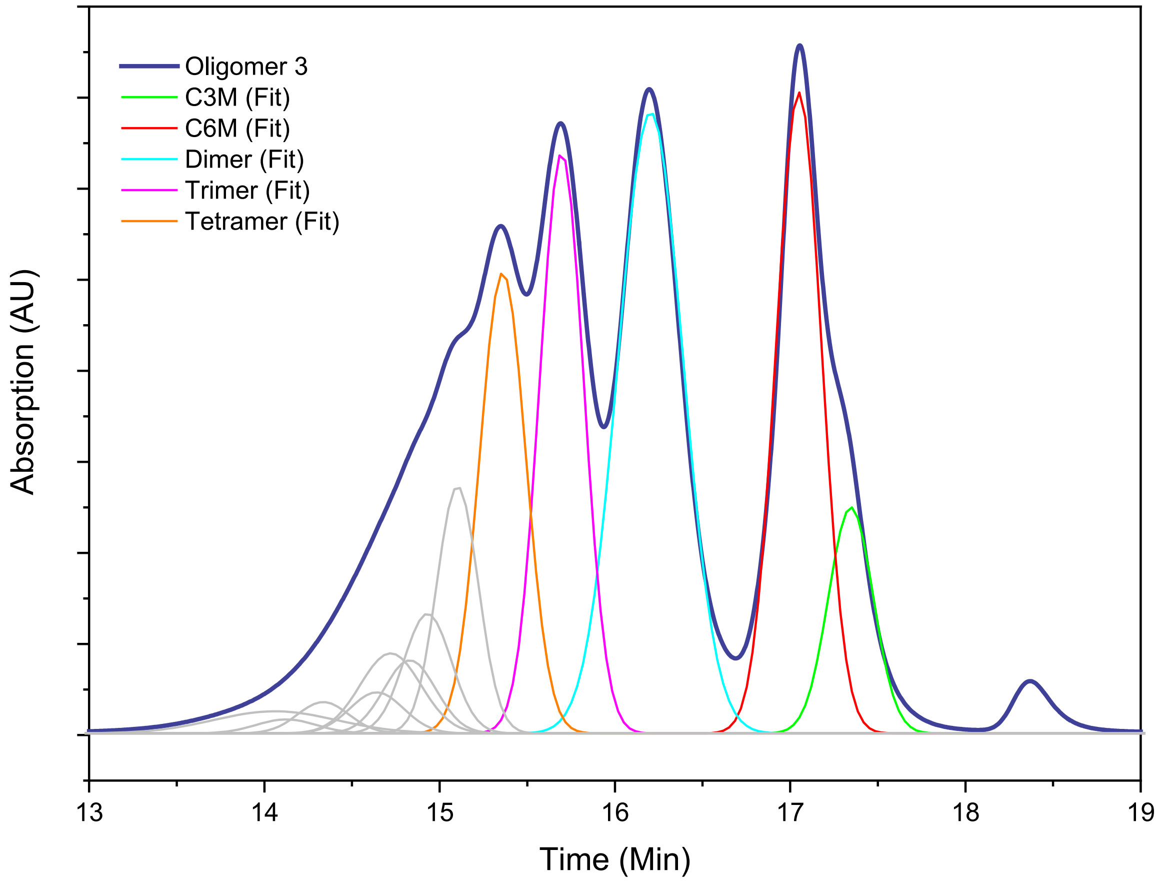

Figure S.6: Deconvoluted GPC spectrum of Oligomer 3. Note that oligomers longer than the tetramer no longer show individual peaks in the original data and are therefore approximated as a set of curves with equal area instead.

Table S.1: Integrated signals corresponding to the deconvoluted GPC spectrum of Oligomer 3

Peak	*Integral (-)*	*Fraction of total (%)*
Monomer C6M	114495,3	17,9
Monomer C3M	39682,4	6,2
Dimer	157776,5	24,7
Trimer	100593,4	15,7
Tetramer	83322,3	13,0
Remainder	143074,7	22,4
TOTAL	638944,5	100

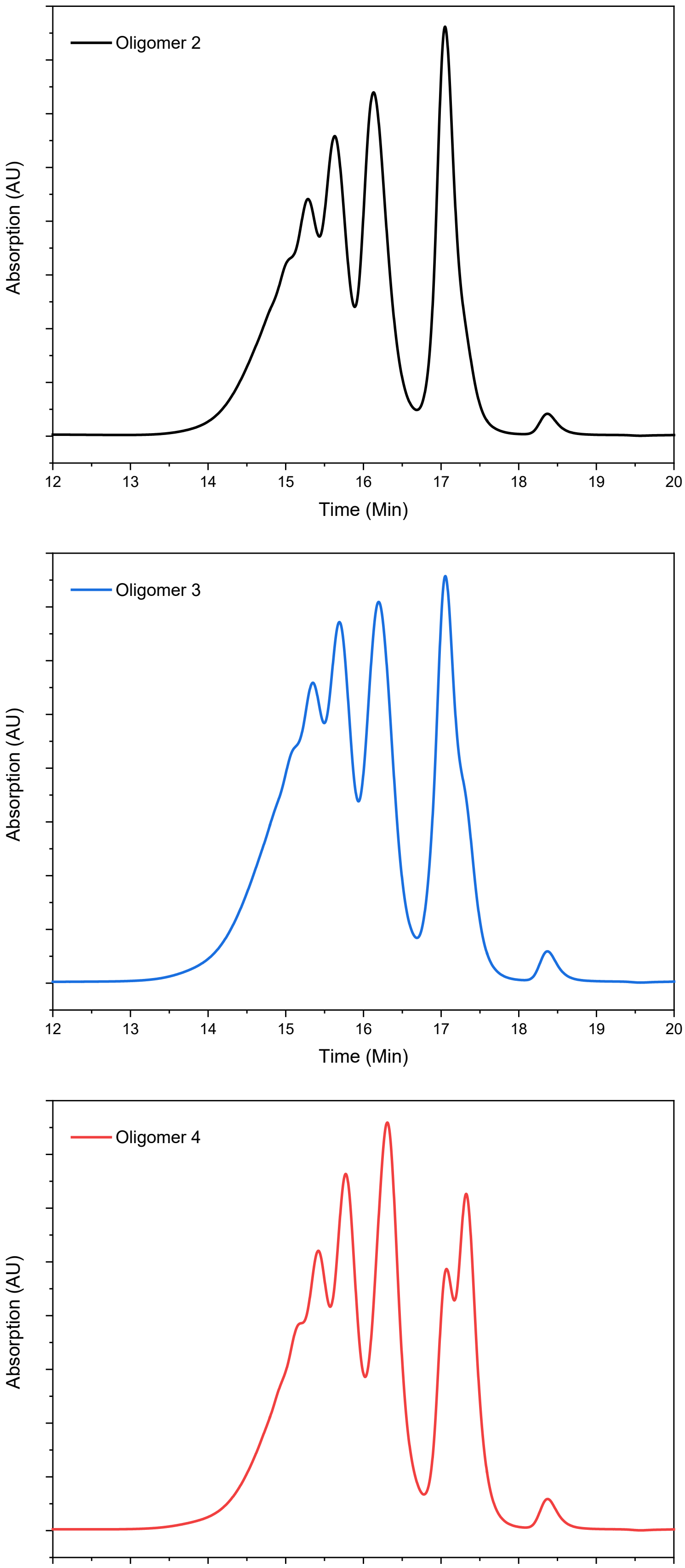

*Figure S.7: GPC profiles of oligomers **2** through **4***

The table below shows the number-average molecular weight (g/mol) corresponding to the major peaks observed in the GPC spectra. The calibration curve used to determine these weights used polystyrene and thus does not reflect their true value, but the general observed trends are reliable and reproducible.

Table S.2: Predicted number-average molecular weights (g/mol) of peaks in GPC profiles, where Peak 1 is the peak corresponding to the monomer(s)

	Peak 1	*Peak 2*	*Peak 3*	*Peak 4*
Oligomer 1	*894*	*2313*	*4032*	*5952*
Oligomer 2	*855*	*2171*	*3770*	*5493*
Oligomer 3	*830*	*2066*	*3554*	*5162*
Oligomer 4	*737*	*1888*	*3270*	*4783*
Oligomer 5	*672*	*1784*	*3115*	*4593*

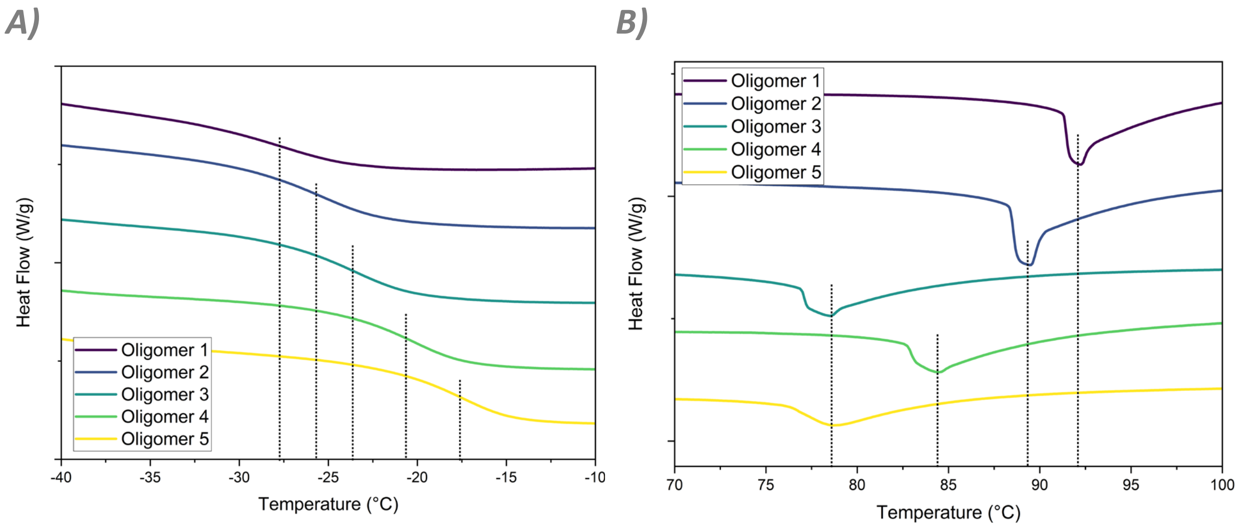

Figure S.8: The data presented shows data vertically adjusted for proper stacking and do not reflect true values of the heat flow. The exotherm is up in all graphs. Dotted lines indicate A) the glass temperature T_g and B) the isotropic transition temperature $T_{Ch,I}$.

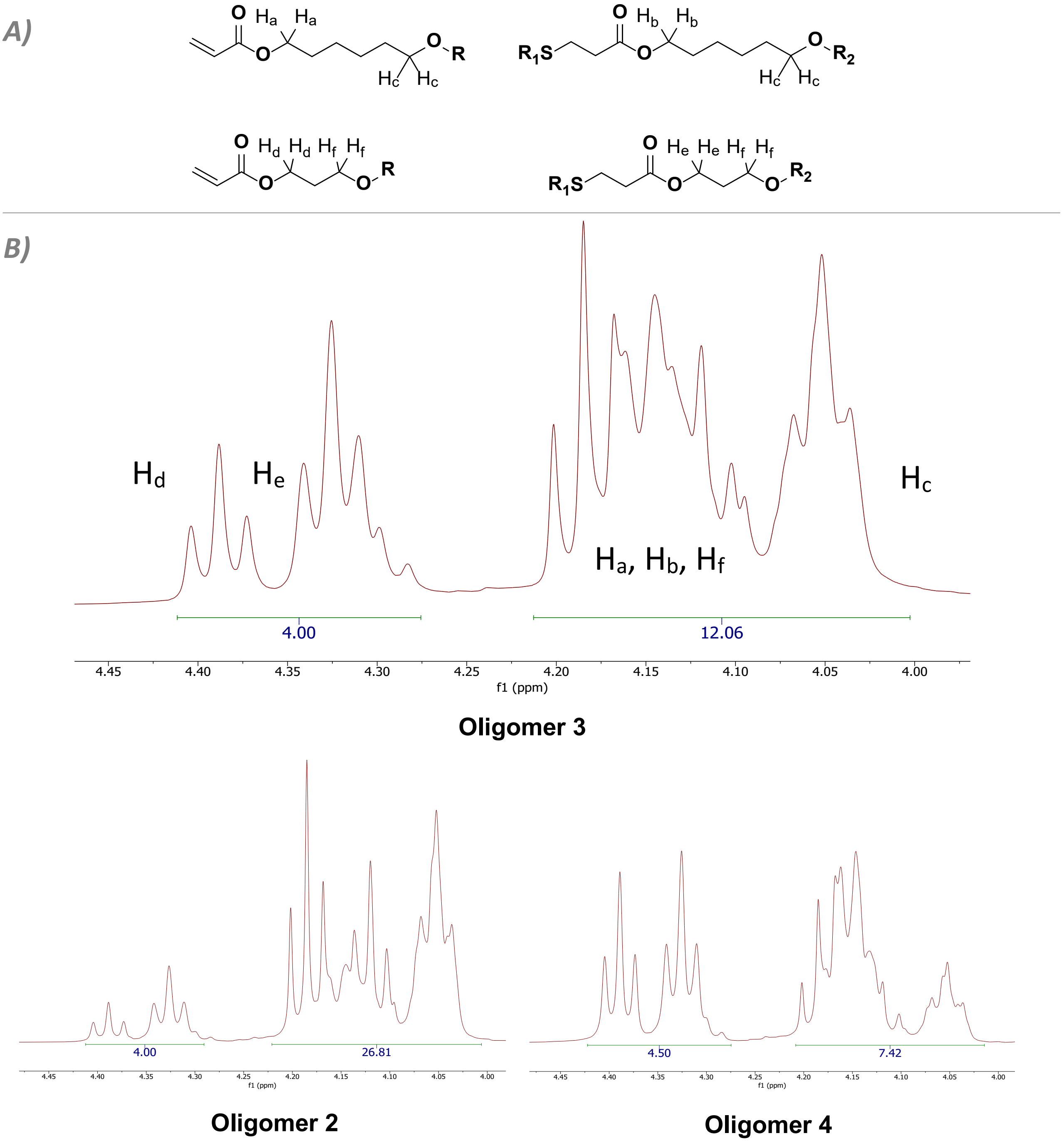

*Figure S.9: A) Partial chemical structures of both reacted and unreacted **C3M** and C6M. B) Part of the ^{1}H-NMR Spectra of oligomers **2**, **3**, and **4** showing the signals corresponding to the hydrogens indicated in A). Values shown demonstrate the relative integrals within the same spectrum.*

The average oligomer **3** should contain one unit of **C6M** and one unit of **C3M**. The integrals corresponding to H_d and H_e are therefore both set to 2. The four remaining groups yield an integral of 12 as shown below. This includes the signal of H_f, so 4 must be subtracted from this integral. This 4, along with the previous 4 from H_d and H_e, shows **C3M** accounts for an integral of 8, leaving the remaining 8 as belonging to **C6M**. Thus, the amounts are equal and the molar feedratio was indeed 1:1. The same logic can be applied to oligomer **2** (8:22.81 or approximately 1:3) and to oligomer **4** (9:2.92 or approximately 3:1).

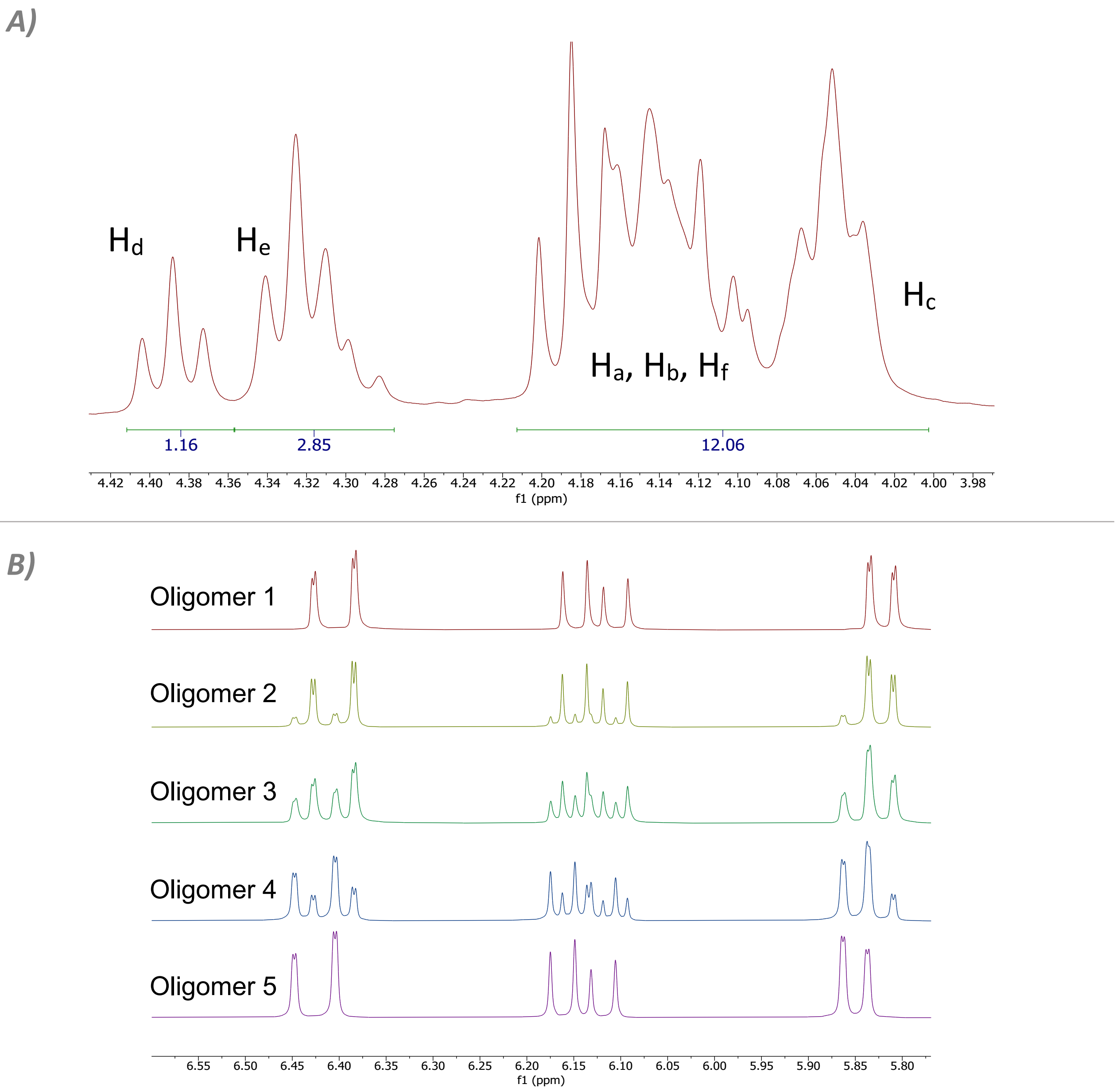

*Figure S.10: NMR-Spectra demonstrating a disproportionate amount of **C3M** reacted compared to **C6M**. A) Partial NMR-spectrum of oligomer **3** as in S.5, with H_d and H_e integrated separately. H_e's integral being greater than H_d's implies there is more reacted than unreacted **C3M** in the mixture. B) Stacked ^{1}H-NMR spectra of all oligomers, showing the signals corresponding to the remaining acrylate groups.*

The following equations are used to numerically determine the relative reaction rates of the two monomers with the dithiol spacer. The contribution of the chiral dopant **4CD** is disregarded.

$$[RSH]_t = [RSH]_{t-\Delta t} - (k_3 * [RSH]_{t-\Delta t} * [C3M]_{t-\Delta t} + k_6/k_3 * [RSH]_{t-\Delta t} * [C6M]_t) * \Delta t$$

$$[C3M]_t = [C3M]_{t-\Delta t} - (k_3 * [RSH]_{t-\Delta t} * [C3M]_{t-\Delta t}) * \Delta t$$

$$[C6M]_t = [C6M]_{t-\Delta t} - (k_6/k_3 * [RSH]_{t-\Delta t} * [C6M]_{t-\Delta t}) * \Delta t$$

$$[C3SR]_t = 1 - [C3M]_t$$

$$[C6SR]_t = 1 - [C6M]_t$$

Here, [RSH] is the relative concentration of dithiol, [C6M] and [C3M] the relative concentrations of both monomers, and [C3SR] and [C6SR] the concentrations of the reaction products of the thiol-ene reaction.

For simplicity k_3 is assumed to be 1. The table below shows the theoretical incorporated acrylate ratios (T.I.A.R.) for different values of k_6/k_3, as well as the true value determined by NMR. These values are then compared to the true values determined by NMR.

Based on this model, the value of k6/k3 is approximately 0.6.

Table S.3: Comparison of the true and theoretical incorporated ratios of C6M and C3M for different values of k6/k3

Oligomer	*Feedratio C6M:C3M*	*Incorporated Ratio C6M:C3M*	*T.I.A.R. (k_6/k_3 = 0.3)*	*T.I.A.R. (k_6/k_3 = 0.4)*	*T.I.A.R. (k_6/k_3 = 0.5)*	*T.I.A.R. (k_6/k_3 = 0.6)*	*T.I.A.R. (k_6/k_3 = 0.7)*
2	*74:26*	*71:29*	*58:42*	*62:38*	*65:35*	*68:32*	*70:30*
3	*50:50*	*40:60*	*30:70*	*34:66*	*38:62*	*41:59*	*44:56*
4	*26:74*	*17:83*	*12:88*	*14:86*	*17:83*	*19:81*	*20:80*

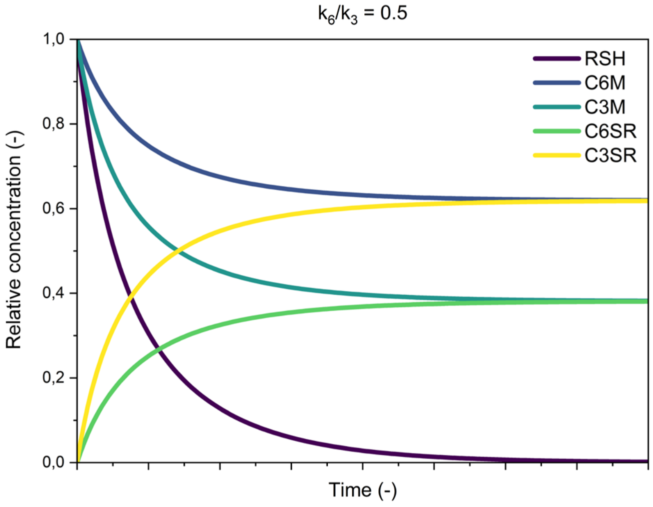

*Figure S.11: Plot showing the theoretical reaction progression for **C6M** and **C3M** when k_6/k_3 equals 0.5.*

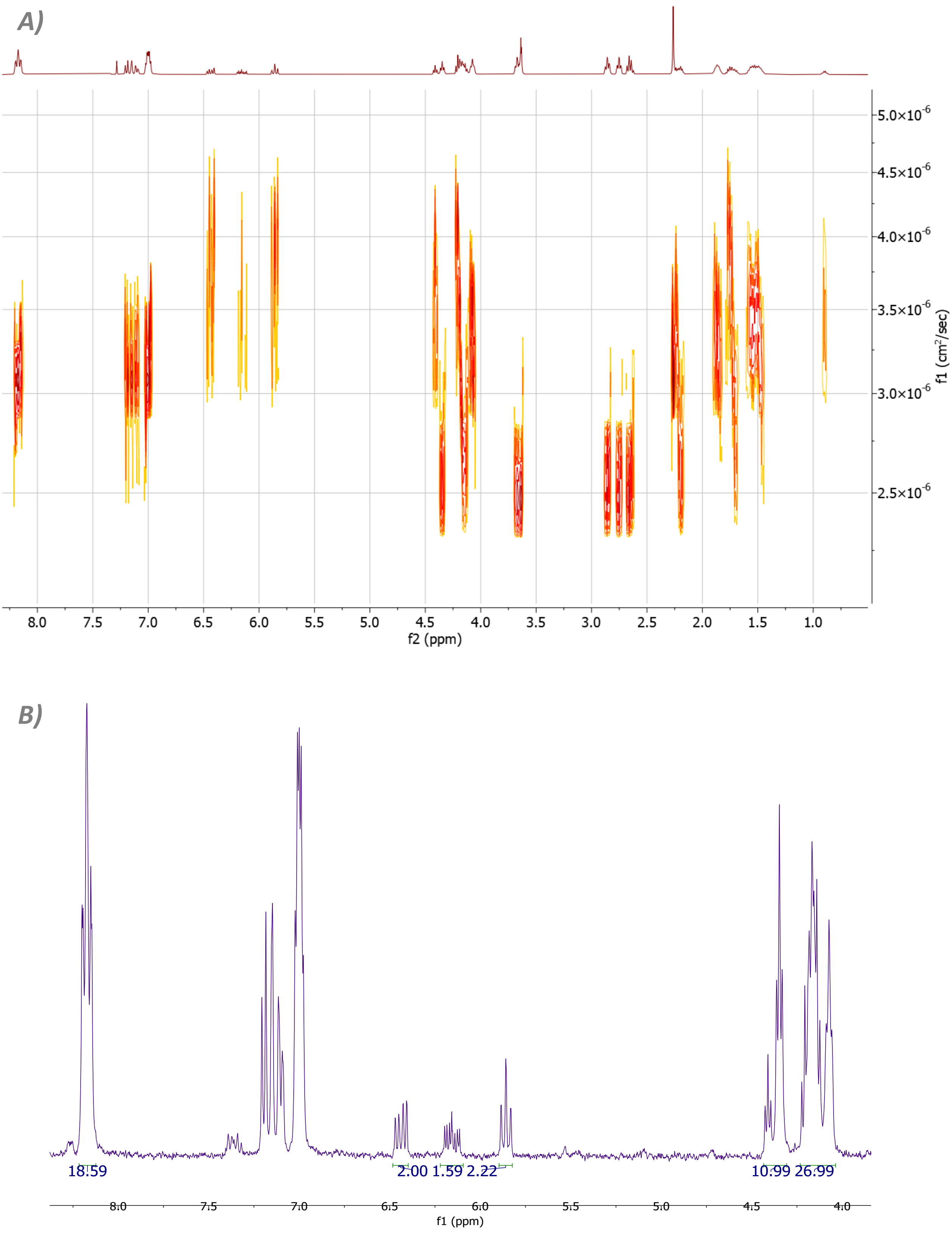

*Figure S.12: A) DOSY-NMR Spectrum of oligomer **3**. B) Extracted spectrum at relatively low diffusion coefficient.*

The formula to determine the chain length described in S.1 results in a DP of 4.8. The peaks between 4.0 and 4.5 ppm can be used analogously to S.8 to yield a ratio of **C3M:C6M** of approximately 22:16 or 1.4:1. Larger oligomers in the mixture thus have more **C3M** incorporated than the average molecule in the mixture.

Table S.4: Series of POM images showing the behavior of coatings 1 and 2 as they cross the smectic-cholesteric transition temperature.

	**Coating 1**	**Coating 2**
**20°C**	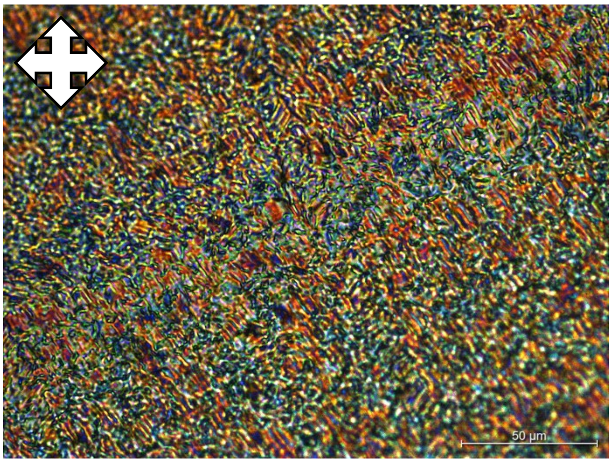	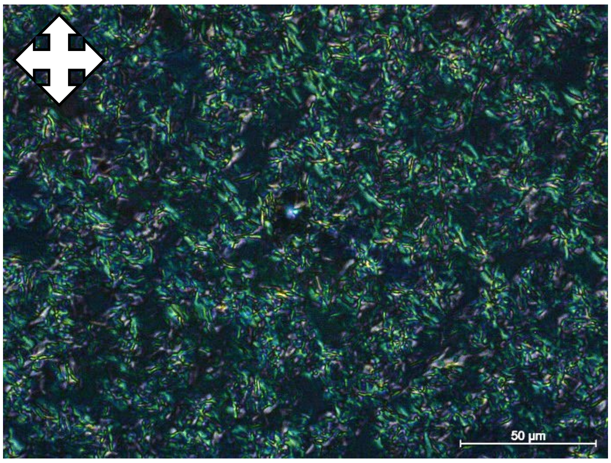
**30°C**	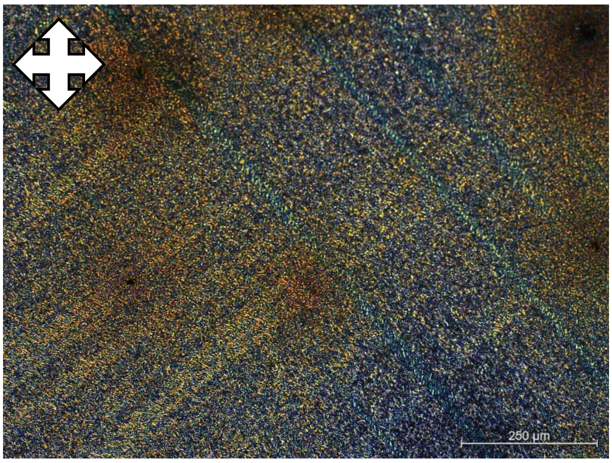	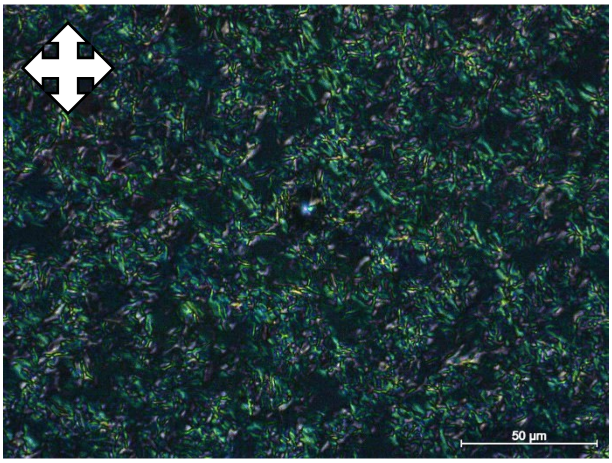
**40°C**	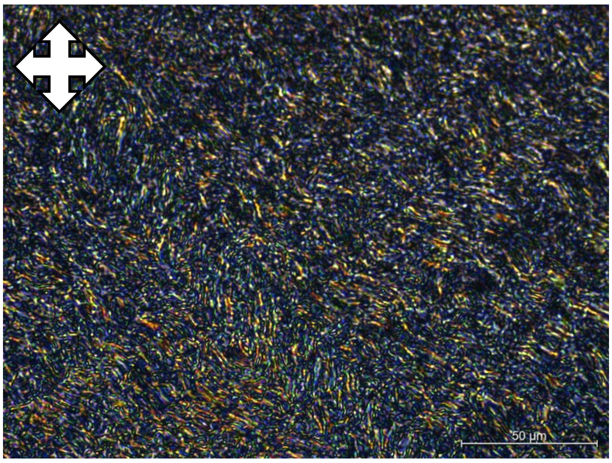	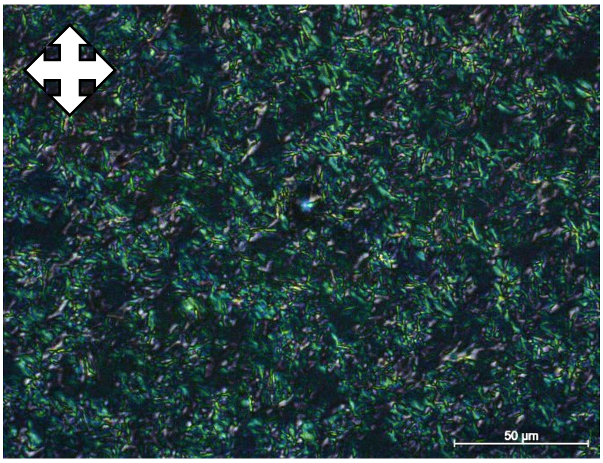

	Coating 1	Coting 2
**50°C**	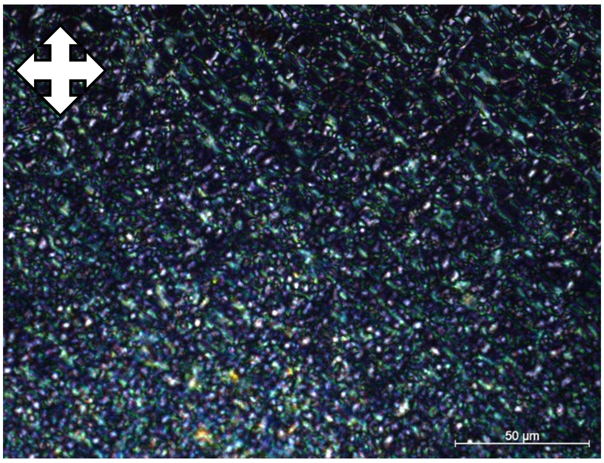	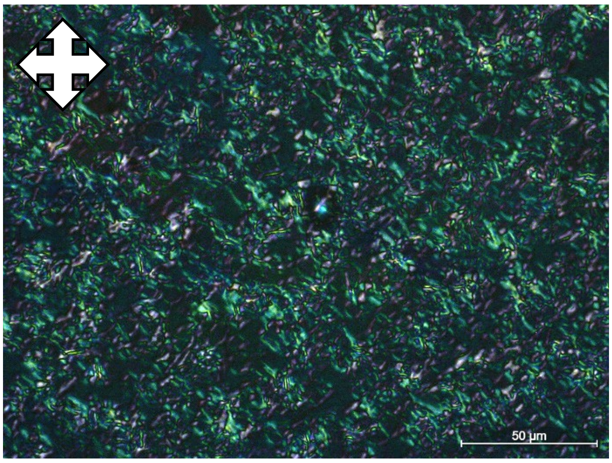
**60°C**	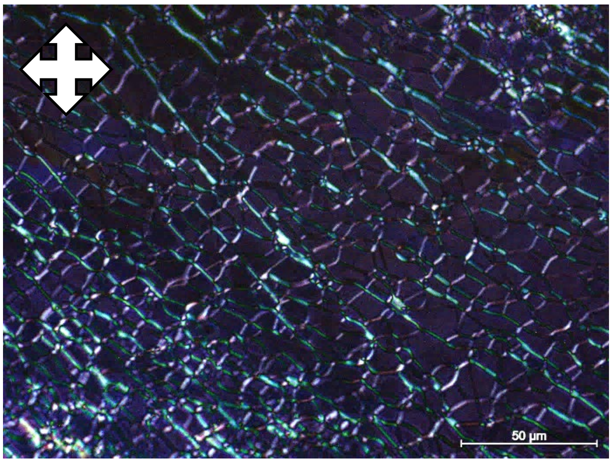	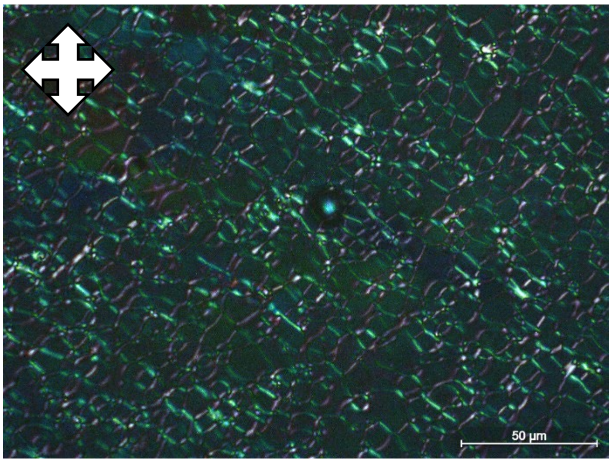
**70°C**	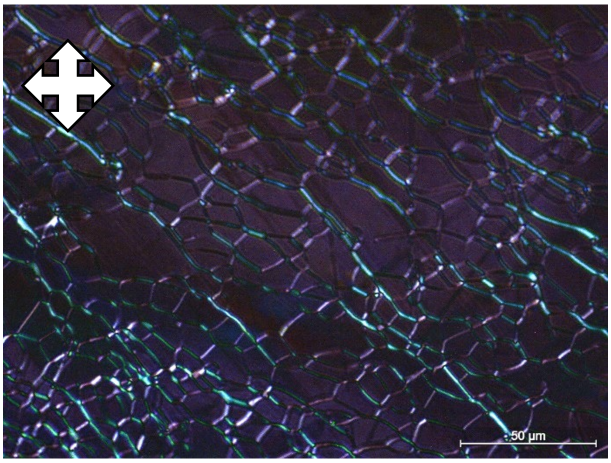	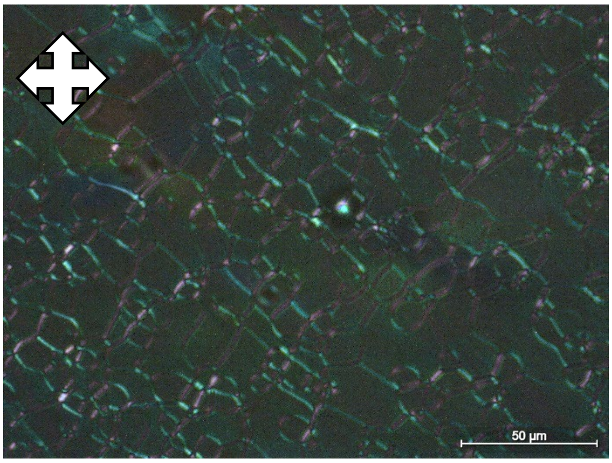

POM Images were taken from 2-month old samples. Crossed arrows on the images indicate the direction of the crossed polarizers. Images had their brightness modified to enhance their visibility.

Oligomer 1

Oligomer 2

Oligomer 3

Oligomer 4

Oligomer 5

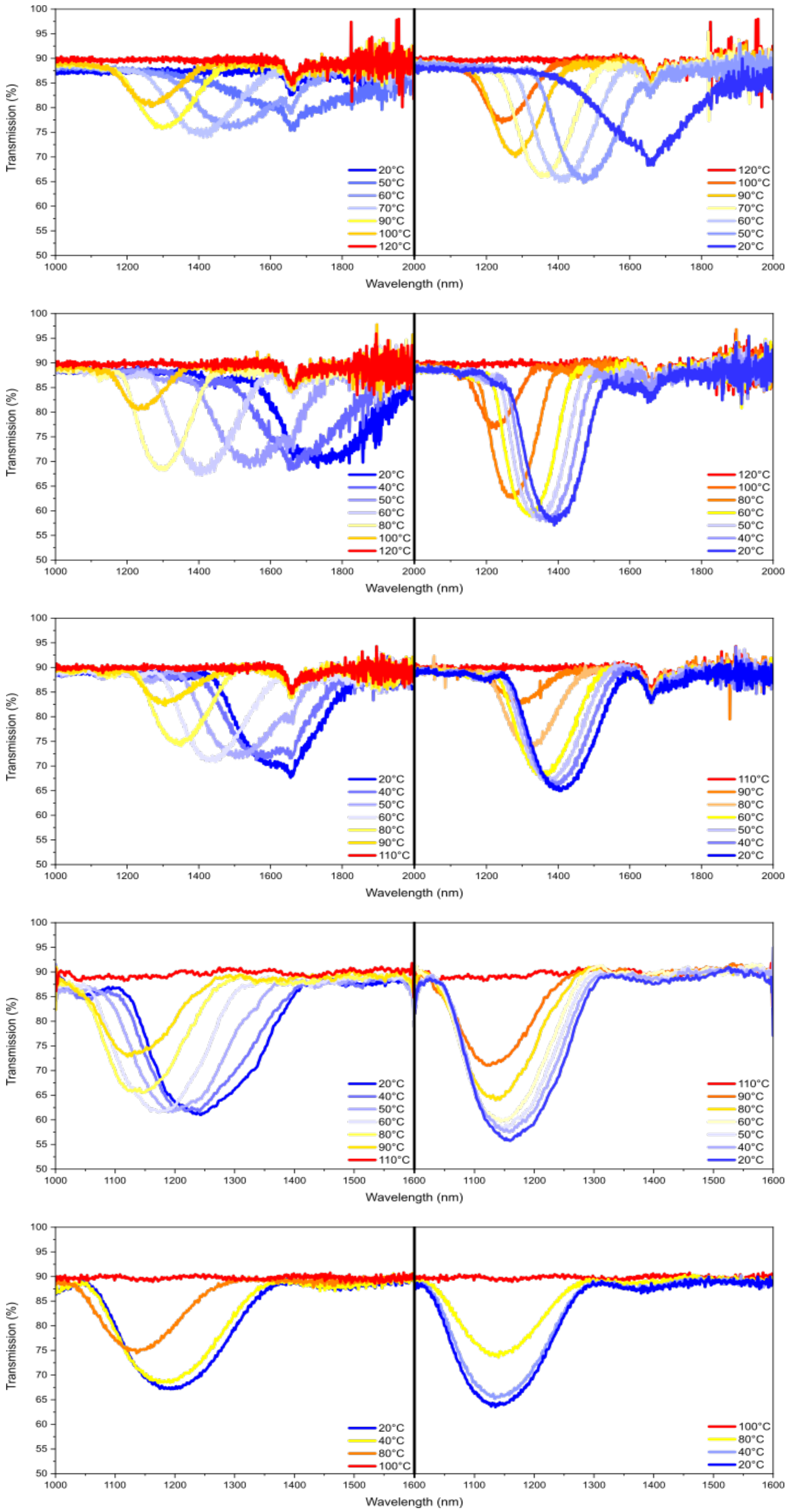

Figure S.13: Temperature-dependent UV-Vis Spectra of Oligomers 1 through 5. Spectra on the left are collected during the heating cycle; spectra on the right are collected during the cooling cycle.

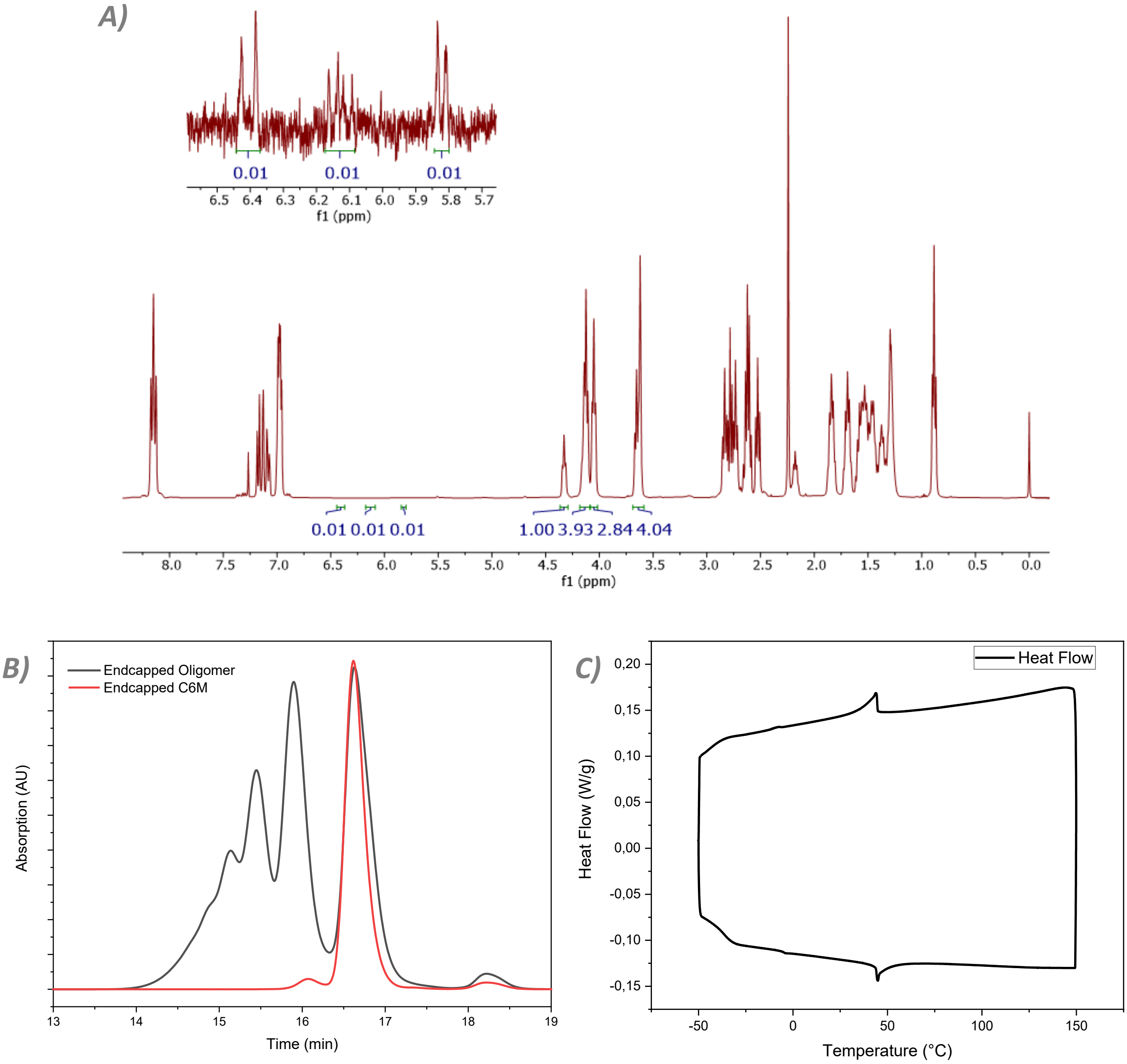

Figure S.14: Characterization of Hexanethiol-endcapped Oligomer 2, showing the A) ^{1}H-NMR spectrum, with an inset showing the trace amounts of remaining acrylate, B) GPC-profile and C) DSC trace.

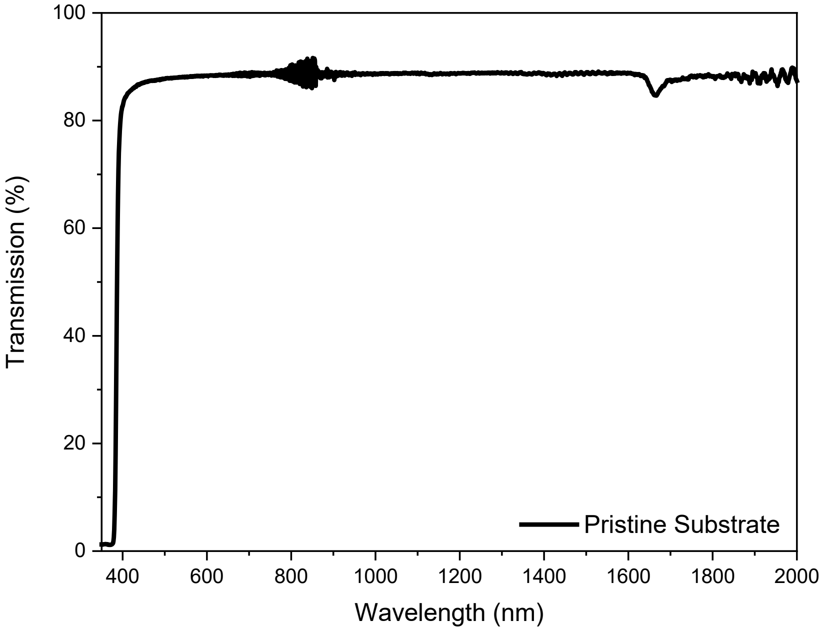

Figure S.15: Transmission spectrum of pristine substrate
